# Structural and functional analysis of betaine aldehyde dehydrogenase from *Staphylococcus aureus*

**DOI:** 10.1107/S1399004715004228

**Published:** 2015-04-25

**Authors:** Andrei S. Halavaty, Rebecca L. Rich, Chao Chen, Jeong Chan Joo, George Minasov, Ievgeniia Dubrovska, James R. Winsor, David G. Myszka, Mark Duban, Ludmilla Shuvalova, Alexander F. Yakunin, Wayne F. Anderson

**Affiliations:** a Department of Biochemistry and Molecular Genetics, Northwestern University, 303 East Chicago Avenue, Chicago, IL 60611, USA; b Center for Structural Genomics of Infectious Diseases (CSGID), Chicago, IL 60611, USA; c Biosensor Tools LLC, Salt Lake City, UT 84103, USA; d Department of Chemical Engineering and Applied Chemistry, University of Toronto, 200 College Street, Toronto, Ontario M5S 3E5, Canada

**Keywords:** betaine aldehyde dehydrogenase, *Staphylococcus aureus*, structural genomics, high-throughput approach, infectious diseases

## Abstract

When exposed to high osmolarity, methicillin-resistant *Staphylococcus aureus* (MRSA) restores its growth and establishes a new steady state by accumulating the osmoprotectant metabolite betaine. Effective osmoregulation has also been implicated in the acquirement of a profound antibiotic resistance by MRSA. Betaine can be obtained from the bacterial habitat or produced intracellularly from choline *via* the toxic betaine aldehyde (BA) employing the choline dehydrogenase and betaine aldehyde dehydrogenase (BADH) enzymes. Here, it is shown that the putative betaine aldehyde dehydrogenase SACOL2628 from the early MRSA isolate COL (*Sa*BADH) utilizes betaine aldehyde as the primary substrate and nicotinamide adenine dinucleotide (NAD^+^) as the cofactor. Surface plasmon resonance experiments revealed that the affinity of NAD^+^, NADH and BA for *Sa*BADH is affected by temperature, pH and buffer composition. Five crystal structures of the wild type and three structures of the Gly234Ser mutant of *Sa*BADH in the apo and holo forms provide details of the molecular mechanisms of activity and substrate specificity/inhibition of this enzyme.

## Abstract 2

The purified putative betaine aldehyde dehydrogenase SACOL2628 from the early methicillin-resistant *S. aureus* COL has betaine aldehyde dehydrogenase activity and is structurally similar to aldehyde dehydrogenases.

## 1. Introduction   

*Staphylococcus aureus* commensally colonizes the skin, mucous membranes and gastrointestinal tract of healthy humans and causes mild to severe infections in hospital settings (DeLeo & Chambers, 2009 ▶; Eady & Cove, 2003 ▶; Jevons, 1961 ▶; Moellering, 2012 ▶; Scheffler *et al.*, 2013 ▶; Wendlandt *et al.*, 2013 ▶). Methicillin-resistant *S. aureus* (MRSA) is the most dangerous strain and its diverse antibiotic resistance is a major global problem (Paulsen *et al.*, 1997 ▶; Cosgrove *et al.*, 2006 ▶; Wenzel, 2004 ▶; Abboud & Waisbren, 1959 ▶; Walsh, 1999 ▶; Otto, 2010 ▶; Gill *et al.*, 2005 ▶). Community-associated MRSA infections are also on the rise (Moellering, 2012 ▶; Wendlandt *et al.*, 2013 ▶) along with reports of animal/livestock-associated MRSA (Fluit, 2012 ▶; Armand-Lefevre *et al.*, 2005 ▶; Cuny *et al.*, 2010 ▶; Pantosti, 2012 ▶), suggesting that MRSA is a food-borne pathogen (Kluytmans, 2010 ▶).

Food-preservation techniques, including the reduction of water activity, have routinely been used to suppress the growth of food-related pathogens (Archer, 1996 ▶; McDowell, 2004 ▶; McMahon *et al.*, 2007 ▶). However, they have also been implicated in triggering the development and spread of antibiotic resistance (Katzif *et al.*, 2003 ▶; Rowan, 1999 ▶; Rickard *et al.*, 2004 ▶). In *S. aureus*, high salt stress increases resistance to penicillin-based antibiotics (Rohrer *et al.*, 2003 ▶; Chambers & Hackbarth, 1987 ▶; Matthews & Stewart, 1984 ▶), represses the expression of virulence determinants (Chan & Foster, 1998 ▶) and affects cell morphology (Vijaranakul *et al.*, 1995 ▶). Uniquely, *S. aureus* tolerates a wide range of salt concentrations on human skin, in foods or in human habitats by regulating cytoplasmic osmolarity through the accumulation of l-proline, choline, taurine and betaine (Eriksen *et al.*, 1995 ▶; Graham & Wilkinson, 1992 ▶; Otto, 2010 ▶). Betaine is the most effective osmolyte that can be imported from the environment, or it can be synthesized from acquired choline by the choline-conversion pathway enzymes in all kingdoms of life (Roessler & Muller, 2001 ▶; Sleator & Hill, 2002 ▶; Lamark *et al.*, 1991 ▶; Kapfhammer *et al.*, 2005 ▶; Cánovas *et al.*, 2000 ▶; Craig, 2004 ▶; Mendum & Smith, 2002 ▶; Graham & Wilkinson, 1992 ▶; Gill *et al.*, 2005 ▶; Gadda & McAllister-Wilkins, 2003 ▶).

Human tissues are rich sources of choline and betaine that can easily be depleted by colonizing and invading pathogenic bacteria (Ueland, 2011 ▶). The choline-conversion pathway of pathogenic bacteria represents a potential target for inhibiting or attenuating bacterial growth. Choline uptake and its oxidation to betaine is necessary for the survival of *Pseudomonas aeruginosa* in the mouse lung infection model (Wargo, 2013 ▶). Choline induces the expression of *cudA* and *cudB* (Rosenstein *et al.*, 1999 ▶), which encode betaine aldehyde dehydrogenase (BADH) and choline dehydrogenase, respectively. Choline inhibits the growth of a *cudA*-deficient *Staphylococcus xylosus* strain. Similar effects of choline have been observed in *Bacillus subtilis* (Boch *et al.*, 1996 ▶). In *S. aureus*, peak choline-transport activity appears to occur at a lower salt concentration than that of betaine transport. Thus, tolerance to a further salt increase would be determined by the influx of betaine, or its production from accumulated choline through the intermediate toxic product betaine aldehyde, and blocking further choline uptake by products of its oxidation (Kaenjak *et al.*, 1993 ▶; Bae *et al.*, 1993 ▶).

BADHs are a group of enzymes from the aldehyde dehydrogenase (ALDH) superfamily (Jackson *et al.*, 2011 ▶) that primarily catalyze the oxidation of betaine aldehyde (BA) to betaine; some BADHs also utilize other substrates (Muñoz-Clares *et al.*, 2010 ▶; Fitzgerald *et al.*, 2009 ▶). In spite of varied substrate specificities, the generally accepted catalytic mechanism of the nicotinamide adenine dinucleotide (phosphate) [NAD(P)^+^]-dependent BADH/ALDH includes four steps (Feldman & Weiner, 1972 ▶). The first step is the formation of a covalent tetrahedral hemithioacetal–enzyme complex. Cofactor binding repositions a conserved catalytic cysteine for nucleophilic attack on the carbonyl of an aldehyde and the adjacent conserved glutamate residue deprotonates the nucleophile. Secondly, a covalent thioester intermediate is generated after a proton is transferred from the first intermediate to oxidized NAD(P)^+^. The third step includes the release of the reduced cofactor, allowing a nucleophilic water to attack the thioester to generate a second tetrahedral intermediate. Finally, an acid product leaves the active site and the cycle can repeat again. BADHs resemble ALDH in structure and are composed of a Rossmann-fold NAD(P)-binding domain, a catalytic (substrate-binding) domain and an oligomerization (bridging) domain (Muñoz-Clares *et al.*, 2010 ▶). Although BADHs have been characterized in *Escherichia coli* (*Ec*BADH; PDB entries 1wnb and 1wnd; Falkenberg & Strøm, 1990 ▶; Gruez *et al.*, 2004 ▶), *B. subtilis* (Boch *et al.*, 1997 ▶), *Halomonas elongata* DSM 3043 (Cánovas *et al.*, 2000 ▶), *Pseudoalteromonas atlantica* T6c (PDB entry 3k2w; New York SGX Research Center for Structural Genomics, unpublished work), *Agrobacterium tumefaciens* (*At*BADH; PDB entry 3r31; New York Structural Genomics Research Consortium, unpublished work), *P. aeruginosa* (*Pa*BADH; PDB entries 2wme, 2wox, 2xdr and 3zqa; González-Segura *et al.*, 2009 ▶; Díaz-Sánchez *et al.*, 2011; A. G. Díaz-Sánchez, L. González-Segura, E. Rudiño-Piñera, A. Lira-Rocha, A. Torres-Larios & R. A. Muñoz-Clares, unpublished work) and in eukaryotic organisms such as *Gadus morhua* (*Gm*BADH; PDB entries 1a4s and 1bpw; Johansson *et al.*, 1998 ▶), *Pisum sativum* (*Ps*BADH; PDB entries 3iwk and 3imj; Tylichová *et al.*, 2010 ▶), *Spinacia oleracae* (*So*BADH; PDB entry 4a0m; Díaz-Sánchez *et al.*, 2012 ▶) and *Solanum lycopersicum* (*Sl*BADH; PDB entries 4i8p, 4i9b and 4i8q; Kopečny *et al.*, 2013 ▶), questions remain about the molecular mechanism of action of BADH.

The sequenced genome of the early MRSA COL isolate revealed the presence of genes encoding osmoprotectant transport systems and choline-conversion pathway enzymes (Gill *et al.*, 2005 ▶). Although osmotic stress response in *S. aureus* may involve alternative pathways (Tsai *et al.*, 2011 ▶), the putative *S. aureus* BADH (*Sa*BADH) may be a key contributing factor to this process. Initial structural analysis of *Sa*BADH provided a platform for in-depth site-directed mutagenesis studies, which identified residues crucial to function and substrate inhibition, and these have been published elsewhere (Chen *et al.*, 2014 ▶). The aim of this study was to provide essential insights into the structure and function of *Sa*BADH. Our initial kinetic and preliminary surface plasmon resonance (SPR) spectroscopy data revealed temperature, pH and buffer effects on the dehydrogenase activity, stability and NAD^+^/NADH affinity of *Sa*BADH. Among the previously functionally characterized *Sa*BADH mutants (Chen *et al.*, 2014 ▶), the Gly234Ser (G234S) mutant, G234S-*Sa*BADH, had a higher affinity for NAD^+^ and decreased substrate inhibition, suggesting the existence of a structural and functional interaction between the NAD-binding and substrate-binding sites (Chen *et al.*, 2014 ▶). Thus, this mutant was of particular interest and its structure determination in the apo and holo forms provides key atomic details on how the G234S substitution affects NAD^+^ binding. The structures of wild-type *Sa*BADH and G234S-*Sa*BADH purified and crystallized with β-mercaptoethanol (BME) reveal that the catalytic Cys289 is oxidized and this chemical modification may affect the affinity of *Sa*BADH for its natural ligands. To maximize the structural resemblance of *Sa*BADH to its native apo and holo states, we determined structures of the enzyme in the absence of BME. Comparison of the BME-free structures with those in the presence of BME highlight some interesting features that provide further understanding of the catalytic and inhibitory potential of *Sa*BADH.

## 2. Materials and methods   

### 2.1. Expression and purification of *Sa*BADH and G234S-*Sa*BADH   

*Sa*BADH was cloned into the pMCSG7 vector and expressed in *E. coli* BL21(DE3)/pMAGIC cells. The G234S mutation was introduced using the QuikChange site-directed mutagenesis kit (Stratagene, La Jolla, California, USA) and the construct was cloned into the p15TV-LIC vector for expression. For each construct, *E. coli* cells harboring the expression plasmid were grown to an OD_600_ of 0.8 at 310 K with aeration at 200 rev min^−1^, chilled to 289 K and induced with 1 m*M* IPTG overnight. Cells were collected (6118*g*, 277 K) and lysed by sonication on ice in 10 m*M* Tris–HCl pH 8.3 buffer, 500 m*M* NaCl, in the presence of 5 m*M* BME (buffer *A*) [*Sa*BADH^BME(+)^ and G234S-*Sa*BADH^BME(+)^] or in the presence of 0.5 m*M* tris(2-carboxyethyl)phosphine (TCEP) (buffer *B*) [*Sa*BADH^BME(−)^ and G234S-*Sa*BADH^BME(−)^]. The proteins were purified using an Ni–NTA column (GE Healthcare, Piscataway, New Jersey, USA) and a Superdex 200 column (pre-equilibrated with either buffer *A* or *B*; GE Healthcare) at 277 K. The purity of the proteins was assayed by SDS–PAGE. The proteins were stored at 193 K.

### 2.2. Enzyme kinetics of *Sa*BADH   

The dehydrogenase activity of *Sa*BADH was determined at 303 K by measuring the continuous absorbance increase of NADH at 340 nm (∊ = 6.22 m*M* ^−1^ cm^−1^). The optimal catalytic pH was determined using 0.15 m*M* betaine aldehyde and 1 m*M* NAD^+^ in various buffers at 100 m*M*: 2-(*N*-morpholino)ethanesulfonic acid (MES; pH 5.5–6.5), potassium phosphate (pH 7.0–8.0) and *N*-cyclohexyl-2-aminoethanesulfonic acid (CHES; pH 8.5–10.0). Kinetic parameters were determined with various concentrations of one substrate in the presence of a saturated or subinhibitory concentration of the second substrate in 100 m*M* potassium phosphate buffer pH 8.0 and were calculated from the initial reaction rates using (1) or (2) with *GraphPad Prism* v.5.02 (GraphPad Software, San Diego, California, USA),
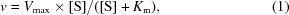


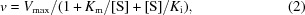
where [S] is the concentration of the varied substrate, *K*_m_ is the Michaelis–Menten constant of the varied substrate and *K*_i_ is the dissociation constant of the varied substrate. One unit (U) of enzyme activity corresponds to the release of 1 µmol of NADH in 1 min. The effect of increasing the ionic strength on betaine aldehyde dehydrogenase activity was tested in the presence of 0.0–2.5 *M* NaCl/KCl with 5 m*M* NAD^+^, 1 m*M* betaine aldehyde and 100 m*M* 4-(2-hydroxyethyl)-1-piperazineethanesulfonic acid (HEPES) buffer pH 8.0.

### 2.3. SPR studies of *Sa*BADH   

*Sa*BADH was studied by SPR using Biacore S51 and 4000 optical biosensors (GE Healthcare). *Sa*BADH (in buffer *A* at 22 mg ml^−1^) was immobilized (0.4 mg ml^−1^ in 10 m*M* sodium acetate buffer pH 5.0) on a CM5 sensor chip (GE Healthcare) using the standard amino-coupling method. Initially, 2 m*M* NAD^+^/NADH (using twofold dilution series; each concentration was tested three times) were probed for interactions with immobilized protein in 10 m*M* phosphate-buffered saline (PBS) buffer pH 7.0 containing 0.1% Brij-35 and 5 m*M* DTT at 277 K. This test revealed a fivefold higher affinity for NADH and thus all further SPR measurements were performed with NADH. The short-term (∼6 h) pH-dependent (pH range 6.0–8.0 in increments of 0.25) strength of the NADH–*Sa*BADH interaction was analyzed using an *Sa*BADH chip from the initial test and 333 µ*M* NADH (threefold dilution series; each concentration was measured twice) at 277 K. The long-term test within the pH range 5.0–8.0 (in increments of 1.0) was conducted with 2 m*M* NADH (threefold dilution series; each concentration was measured twice) over 2 d starting with freshly immobilized *Sa*BADH at 298 K. The pH dependence of NADH binding to *Sa*BADH was compared with that of adenosine diphosphate (ADP; both ligands at 2 m*M*; pH range 5.0–8.0 at 298 K) using two-day-old immobilized *Sa*BADH. Buffer-composition effects on the affinity of *Sa*BADH for NADH were assayed in 10 m*M* buffers: (i) HEPES-buffered saline (HBS), (ii) potassium bisulfite (KBS) and (iii) 2-amino-2-hydroxymethyl-propane-1,3-diol (Tris)-buffered saline (TBS) at pH 7.0 and 298 K. Threefold dilution series starting at 2 m*M* NADH were used, with each concentration tested twice. Binding of betaine and BA (both 1 m*M*) to *Sa*BADH was tested in 20 m*M* sodium phosphate buffer pH 7.0, 150 m*M* NaCl, 0.1% Brij-35, 2 m*M* DTT, 5% DMSO. The *Salmonella typhimurium* LT2 3-dehydroquinate dehydratase (AroD; Light *et al.*, 2013 ▶) and its substrate dehydroquinic acid (DQA; 200 µ*M*) were used as negative controls. Sodium malonate was probed for binding as a double-negative control for both AroD and *Sa*BADH. Binding responses, measured as resonance units (RU), were double referenced and fitted with the Langmuir binding model using *SCRUBBER*2 available at http://www.biologic.com.au.

### 2.4. Size-exclusion chromatography with multi-angle laser-light scattering (SEC-MALS)   

*Sa*BADH^BME(+)^ was run on a Superdex 200 10/300 GL column (GE Healthcare) to estimate its molecular mass and oligomerization state as described previously (Halavaty *et al.*, 2012 ▶). *Sa*BADH^BME(+)^ (2 mg ml^−1^) was run in 10 m*M* Tris–HCl pH 8.3, 500 m*M* NaCl, 5 m*M* BME on a the column pre-equilibrated with the same buffer at a flow rate of 0.4 ml min^−1^ at 295 K. Bovine serum albumin (BSA; Sigma–Aldrich, St Louis, Missouri, USA) was used as a control protein. The molecular weights of the proteins were determined using the *ASTRA* software (Wyatt Technology Europe, Dernbach, Germany).

### 2.5. Dynamic light scattering (DLS)   

All solutions were centrifuged and filtered prior to DLS analysis of *Sa*BADH^BME(−)^. A Zetasizer Nano S instrument (Malvern Instruments Inc., Westborough, Massachusetts, USA) and *Zetasizer* software 7.01 were used to perform measurements and determine the *Z*-average hydrodynamic radius (*R*_h_), polydispersity index (PdI) and molecular weight (MW) using a globular polymer model. Data were acquired (ten acquisitions of 5 s each for each of the *Sa*BADH concentrations) at 298 K using auto-attenuated He–Ne laser power (wavelength 663 nm) and a solvent refractive index of 1.33. *Sa*BADH was tested at 10, 20, 30, 50, 80 and 100 µ*M* in 10 m*M* Tris–HCl buffer pH 8.3, 0.5 m*M* TCEP and supplemented with 0.0, 0.5, 1.0, 1.5, 2.0 or 2.5 *M* NaCl or KCl. The analyzed data are an average of triplicate measurements over all concentrations of NaCl or KCl.

### 2.6. Crystallization of *Sa*BADH and G234S-*Sa*BADH   

Proteins were crystallized using the sitting-drop vapor-diffusion technique, a Phoenix protein crystallization robot (Art Robbins Instruments, Sunnyvale, California, USA) and crystallization screens from Qiagen (Valencia, California, USA) at 295 K. Apo *Sa*BADH^BME(+)^ (at 7.4 mg ml^−1^) and apo *Sa*BADH^BME(−)^ (at 7.0 mg ml^−1^) were crystallizaed using a condition from The JCSG+ Suite consisting of 200 m*M* MgCl_2_, 100 m*M* Tris pH 8.5, 20%(*w*/*v*) PEG 8000. Crystals of apo *Sa*BADH^BME(+)^ with a modeled PEG molecule at the NAD- and substrate-binding sites [apo *Sa*BADH^BME(+)PEG^] was obtained from a condition from The Classics II Suite consisting of 100 m*M* citric acid pH 3.5, 25%(*w*/*v*) PEG 3350. NAD–*Sa*BADH^BME(+)^ (at 7.4 mg ml^−1^ in 10 m*M* Tris–HCl pH 8.3, 250 m*M* NaCl, 5 m*M* BME plus 2 m*M* NAD^+^) was crystallized using a condition from The PEGs II Suite consisting of 200 m*M* sodium acetate, 100 m*M* Tris pH 8.5, 30%(*w*/*v*) PEG 4000. NAD–*Sa*BADH^BME(−)^ and NAD–G234S-*Sa*BADH^BME(−)^ (both at 7.0 mg ml^−1^ plus 2 m*M* NAD^+^) were crystallized using a condition from The JCSG+ Suite consisting of 100 m*M* potassium thiocyanate, 30%(*w*/*v*) PEG 2000 MME and a condition from The Classics II Suite consisting of 200 m*M* ammonium acetate, 100 m*M* Tris pH 8.5, 25%(*w*/*v*) PEG 3350, respectively. Apo G234S-*Sa*BADH^BME(−)^ (7.0 mg ml^−1^) and apo G234S-*Sa*BADH^BME(+)^ (at 7.3 mg ml^−1^) were crystallized using conditions from The PACT Suite consisting of 100 m*M* sodium propionate, sodium cacodylate and bis-tris propane (PCB) buffer pH 8.0, 25%(*w*/*v*) PEG 1500 and 100 m*M* succinic acid, sodium dihydrogen phosphate and glycine (SPG) buffer pH 8.0, 25%(*w*/*v*) PEG 1500, respectively. Crystals were soaked in the respective crystallization solutions for cryoprotection and flash-cooled in liquid nitrogen prior to X-ray data collection.

### 2.7. X-ray data collection, structure determination and validation   

X-ray diffraction data were collected on beamlines 21-ID-F, 21-ID-G and 21-ID-D at the Life Science Collaborative Access Team (LS-CAT), Advanced Photon Source (APS), Argonne National Laboratory (ANL) (Tables 1 ▶ and 2 ▶). Data sets were processed with *HKL*-2000 (Otwinowski & Minor, 1997 ▶) and *HKL*-3000 (Minor *et al.*, 2006 ▶). The crystal structure of apo *Sa*BADH^BME(+)^ was determined by molecular replacement using a single chain of the human mitochondrial ALDH (PDB entry 1cw3; Ni *et al.*, 1999 ▶) as a model and *Phaser* (McCoy *et al.*, 2007 ▶) from the *CCP*4 package (Winn *et al.*, 2011 ▶). The initial structure solution was rebuilt with *ARP*/*wARP* (Morris *et al.*, 2003 ▶), manually modified in *Coot* (Emsley & Cowtan, 2004 ▶; Emsley *et al.*, 2010 ▶) and refined with *REFMAC* v.5.7 (Murshudov *et al.*, 2011 ▶). The other *Sa*BADH and G234S-*Sa*BADH crystal structures were determined by molecular replacement using the apo *Sa*BADH^BME(+)^ structure (PDB entry 4mpb) as a model. Translation–libration–screw (TLS) groups were obtained from the *TLS Motion Determination* (*TLSMD*) server (Painter & Merritt, 2006 ▶; http://skuld.bmsc.washington.edu/~tlsmd/) and introduced at the final stages of refinement. The quality of the structures was checked with the PDB validation server (http://deposit.pdb.org/validate/) and *MolProbity* (Chen *et al.*, 2010 ▶; Davis *et al.*, 2007 ▶; http://molprobity.biochem.duke.edu/). Diffraction images for the deposited structures are available at the Center for Structural Genomics of Infectious Diseases (CSGID) website (http://www.csgid.org/csgid/pages/home). The pairwise structural alignments were produced with the *DaliLite* server (Holm & Park, 2000 ▶; http://www.ebi.ac.uk/Tools/structure/dalilite/). The total buried surface area of the *Sa*BADH assemblies was determined using the *Protein Interfaces, Surfaces and Assemblies* (*PISA*) service at the European Bioinformatics Institute (http://www.ebi.ac.uk/pdbe/prot_int/pistart.html; Krissinel & Henrick, 2007 ▶). Structural figures were generated with *PyMOL* (Schrödinger) and *LigPlot*+ v.1.4 (Laskowski & Swindells, 2011 ▶). Data-collection and structure-determination statistics are given in Table 1 ▶ for *Sa*BADH and in Table 2 ▶ for G234S-*Sa*BADH. The coordination geometries of modeled Na^+^, K^+^ and Mg^2+^ were validated with the *CheckMyMetal* (*CMM*) server (http://csgid.org/csgid/metal_sites/; Zheng *et al.*, 2014 ▶). The identity of K^+^ was also confirmed by calculating an anomalous difference Fourier map.

## 3. Results   

### 3.1. Enzymatic activity of *Sa*BADH   

**Figure 1:**
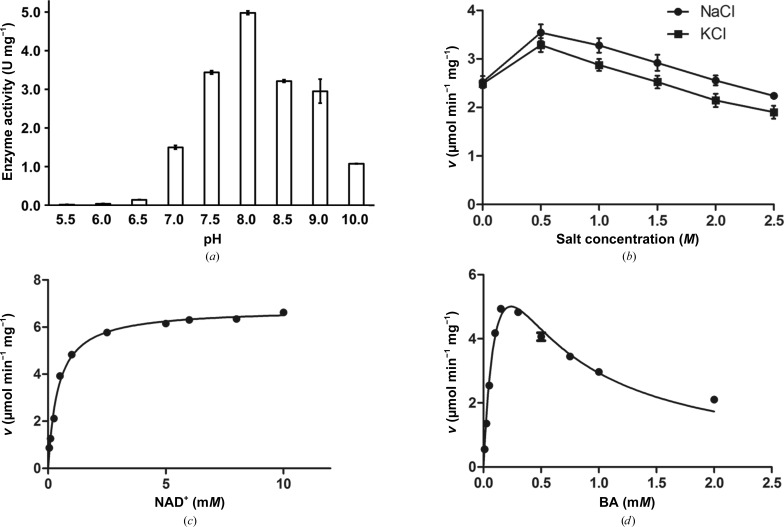
BADH activity of *Sa*BADH. (*a*) pH dependence of the *Sa*BADH activity. (*b*) Ionic strength dependence of the *Sa*BADH activity. (*c*, *d*) The Michaelis–Menten kinetics of *Sa*BADH. BA, betaine aldehyde.

Based on its sequence, the uncharacterized protein SACOL2628 from *S. aureus* was annotated as a BADH (*Sa*BADH; UniProtKB entry Q5HCU0). The purified recombinant protein was highly specific for BA (Fig. 1 ▶) and showed negligible activity against other aldehyde substrates (Chen *et al.*, 2014 ▶). *Sa*BADH was also active with NAD(P)^+^, but the NAD-supported activity was at least ten times higher (Fig. 1 ▶, Table 3 ▶). The protein exhibited maximal activity at 500 m*M* NaCl/KCl and pH 8.0 and was fairly resistant to increasing ionic strength (Fig. 1 ▶). *Sa*BADH had negligibly slower kinetics in the presence of NAD^+^/K^+^ *versus* NAD^+^/Na^+^ (Fig. 1 ▶ *b*), suggesting a functional preference for sodium. *Sa*BADH was strongly inhibited by BA at concentrations of higher than 0.15 m*M* (Fig. 1 ▶ *d*, Table 3 ▶), similarly to other BADHs (Velasco-García *et al.*, 1999 ▶, 2000 ▶; Falkenberg & Strøm, 1990 ▶; Valenzuela-Soto & Muñoz-Clares, 1994 ▶; Boch *et al.*, 1997 ▶). Detailed kinetic and inhibition studies of *Sa*BADH have been published elsewhere (Chen *et al.*, 2014 ▶).

### 3.2. SPR: *Sa*BADH binds NAD^+^(H) in a pH-dependent manner   

**Figure 2:**
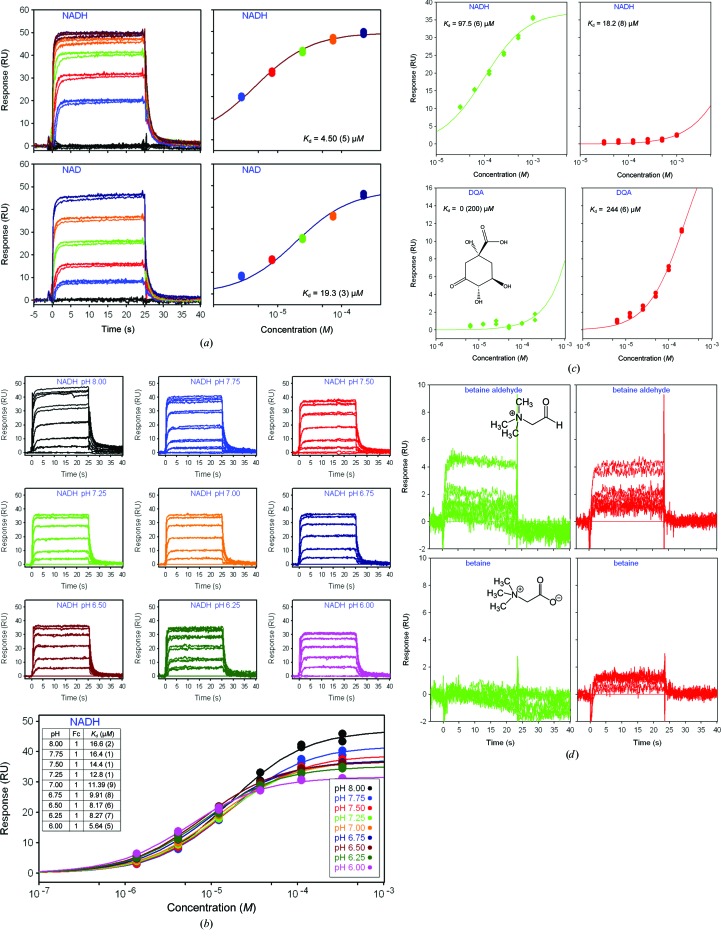
The affinity of *Sa*BADH for ligands as studied by SPR. (*a*) Relative response and single binding isotherm fitting for NAD^+^ and NADH binding to the enzyme at pH 7.0 and 277 K. (*b*) Response and data fitting of the NADH–*Sa*BADH interactions in the pH range 6.0–8.0 at 277 K. (*c*) Single binding isotherm fitting of the affinity of 3-dehydroquinic acid and NADH for *Sa*BADH (green) and *S. typhimurium* LT2 3-dehydroquinate dehydratase (red) at 298 K and pH 7.0. (*d*) Affinity of immobilized *Sa*BADH (green) and *S. typhimurium* LT2 3-dehydroquinate dehydratase (red) for the betaine aldehyde and betaine at 298 K and pH 7.0.

**Figure 3:**
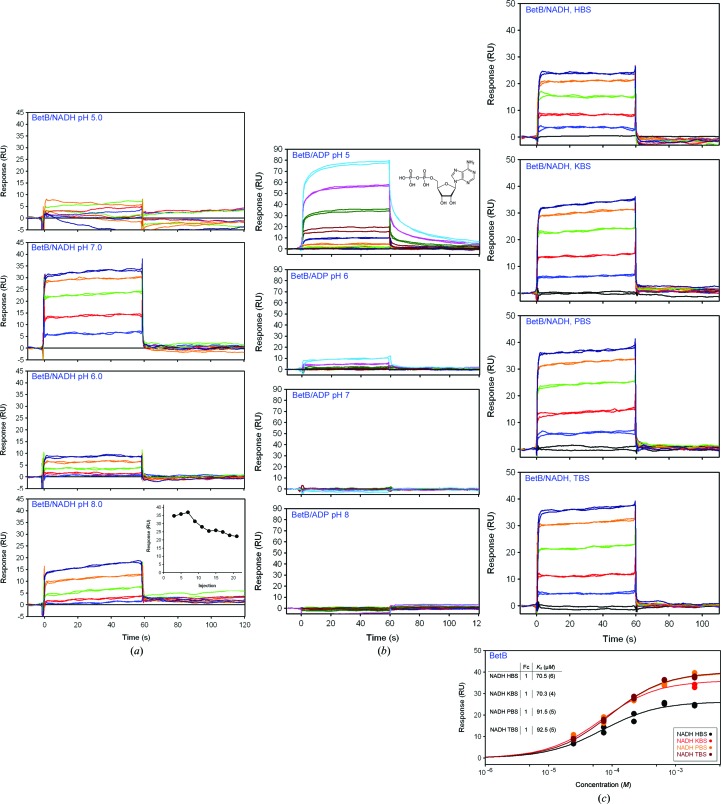
Long-term stability and pH-dependent affinity of *Sa*BADH (BetB) for NADH at 298 K. (*a*) SPR signal showing the pH dependence of NADH binding to a fresh *Sa*BADH surface. No changes in RU were detected using two-day-old immobilized *Sa*BADH at any of the pH values except pH 8.0. The inset illustrates the loss of affinity over 12 h using freshly immobilized *Sa*BADH at pH 8.0 and 298 K. (*b*) pH effect on the interaction of *Sa*BADH with ADP assayed on a two-day-old *Sa*BADH surface at 298 K. (*c*) Relative response and single binding isotherm fitting of NADH (2 m*M*) binding to *Sa*BADH in 10 m*M* HBS, KBS, PBS and TBS buffers at pH 7.0 and 298 K.

**Figure 4:**
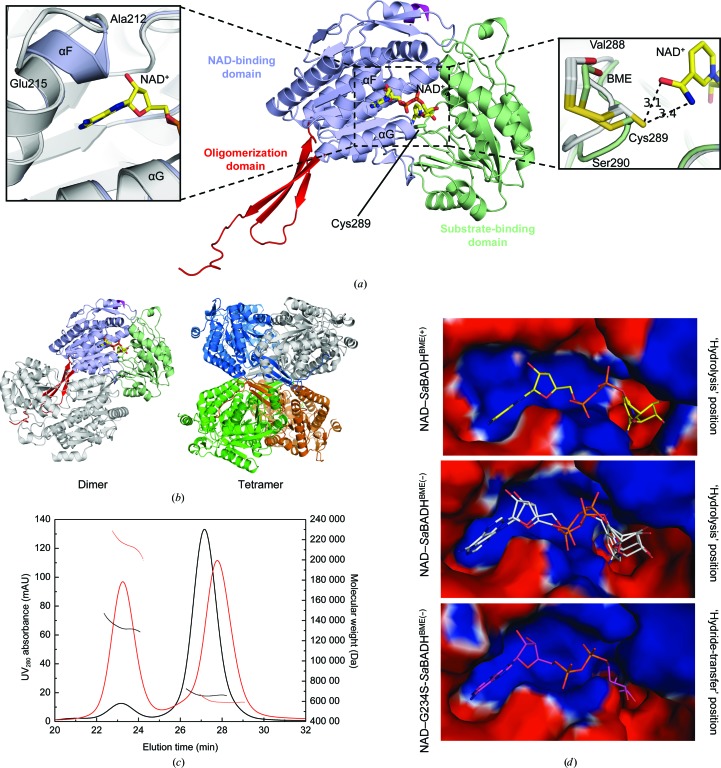
Structure of *Sa*BADH. (*a*) Ribbon representation of the NAD–*Sa*BADH^BME(+)^ structure. NAD and the catalytic Cys289 are shown in stick representation. The insets show key structural differences between the apo structures (gray ribbons) and the NAD-bound structures. (*b*) The quaternary structure of *Sa*BADH. (*c*) SEC-MALS elution profiles of BSA (black) and apo *Sa*BADH^BME(+)^ (red). Molecular-mass distribution curves at 500 m*M* NaCl [BSA, horizontal short black; apo *Sa*BADH^BME(+)^, red] are shown. (*d*) The surface electrostatic potentials (−4.0*kT* e^−1^, red; +4.0*kT* e^−1^, blue) at the NAD-binding site.

NADH was chosen as an indicator of specific binding because of its higher affinity for *Sa*BADH^BME(+)^ (*K*_d_ = 4.50 µ*M*) *versus* NAD^+^ (*K*_d_ = 19.30 µ*M*) (Fig. 2 ▶ *a*) at pH 7.0. The same immobilized *Sa*BADH was further assayed to determine the strength of the NADH–*Sa*BADH interactions in a 6 h pH-dependent experiment at 277 K (Fig. 2 ▶ *b*). From this experiment, a *K*_d_ value of 11.4 µ*M* was obtained at pH 7.0, suggesting that the *K*_d_ differences may be attributed to variations in the experimental setups (see §[Sec sec2]2). The *K*_d_ for NADH at 298 K is higher than at 277 K at pH 7.0 (Figs. 2 ▶ *a* and 2 ▶ *c*), while no differences in the NADH binding were detected at pH 5.0, 6.0 and 7.0 with freshly made and two-day-old *Sa*BADH surfaces at 298 K. However, a slight decrease in RU was observed at pH 8.0 with a two-day-old chip (not shown). Subjecting freshly immobilized *Sa*BADH (tested with 1 m*M* NADH) to a 12 h stability evaluation at pH 8.0, we observed a ∼10 RU decrease from the zero-hour measurement (Fig. 3 ▶ *a*, inset in the pH 8.0 graph). *Sa*BADH and the *S. typhimurium* LT2 3-dehydroquinate dehydratase AroD interacted with sodium malonate (double-negative control; data not shown) nonselectively, while each of the proteins selectively responded to its natural ligand, NADH and DQA, respectively (Fig. 2 ▶ *c*). The two enzymes had very weak affinity or no affinity for both 1 m*M* BA and betaine, as indicated by comparable low-level to noise-level RU signals, respectively (Fig. 2 ▶ *d*). The pH-dependent strength of the NADH–*Sa*BADH interaction was further compared with that between ADP and *Sa*BADH (Fig. 3 ▶ *b*). An acidic pH favored the ADP–*Sa*BADH interaction (Fig. 4 ▶ *d*), while NADH bound more strongly at pH 7.0 and more weakly at pH 8.0 (Fig. 3 ▶ *a*). A moderate decrease in the NADH affinity was observed in the HEPES buffer and was likely to be caused by HEPES binding at the NAD-binding site (Fig. 3 ▶ *c*), as has been observed with other proteins that bind nucleotide-based ligands (Majorek *et al.*, 2014 ▶).

### 3.3. Crystal structure of wild-type *Sa*BADH and G234S-*Sa*BADH   

We previously showed (Chen *et al.*, 2014 ▶) that the substitution of Gly234, which is located in the NAD-binding area, by a serine residue increased the affinity of *Sa*BADH for NAD^+^ and made the mutant more active than the wild type. The mutation also reduced the substrate inhibition. To fully understand the kinetic and inhibition mechanisms of G234S-*Sa*BADH, we determined its crystal structures in apo and holo forms and in the presence/absence of BME and compared them with the wild-type structures.

All eight structures of *Sa*BADH are similar to each other (r.m.s.d. of 0.2–0.8 Å over 496 C^α^ atoms) and the homologous ALDH/BADH (Fig. 4 ▶ *a*). *DALI* (Holm & Rosenström, 2010 ▶) analysis found a putative ALDH from *Burkholderia cenocepacia* (PDB entry 4o6r; Seattle Structural Genomics Center for Infectious Disease, unpublished work) to be the closest structural homolog of *Sa*BADH (*Z*-score of 59.6; r.m.s.d. of 1.3 Å; 38% sequence homology).

Consistent with the high overall similarity, obvious differences between the eight *Sa*BADH structures are confined to residues Ala212–Glu217 (helix αF) and Val288–Ser290 (insets in Fig. 4 ▶ *a*). The first peptide stretch adopts a helical conformation extending helix αF in the presence of NAD^+^ and interacts with the adenine ring of the cofactor. Although this segment is unwound in apo *Sa*BADH^BME(+/−)^, it forms a similar helix turn in apo G234S-*Sa*BADH^BME(+/−)^ and apo *Sa*BADH^BME(+)PEG^. NAD^+^ binding results in displacement of the C^α^ atoms of the second area by ∼1.6–1.9 Å. However, in apo G234S-*Sa*BADH^BME(−)^ and apo *Sa*BADH^BME(+)PEG^ Val288–Ser290 is also repositioned in an NAD-dependent manner.

In the crystal, *Sa*BADH forms a tetramer (∼21 400 Å^2^ buried surface area) that is composed of two dimers with ∼7900 Å^2^ buried surface area each (Fig. 4 ▶ *b*). Hydrogen-bond and van der Waals interactions stabilize the tetramer. SEC-MALS analysis revealed two *Sa*BADH peaks (theoretical monomer mass of 57 360.7 Da with the purification tag) with apparent molecular masses of ∼60 and ∼215 kDa, approximately corresponding to a monomer and tetramer, respectively (Fig. 4 ▶ *c*). DLS analysis was performed to assess the oligomerization state of *Sa*BADH in the presence of 0.5–2.5 *M* NaCl/KCl. In the absence of each salt, *Sa*BADH appears to be predominantly dimeric, with an estimated molecular mass of 168.1 ± 0.2 kDa (*R*_h_ of 5.32 ± 0.27 nm and PdI of 0.25 ± 0.03). An average *R*_h_ of 6.58 ± 1.47 nm (PdI of 0.23 ± 0.14) with an estimated molecular mass of 279.0 ± 8.7 kDa and an average *R*_h_ of 5.27 ± 0.65 nm (PdI of 0.14 ± 0.08) and molecular mass of 164.5 ± 1.2 kDa were obtained for all tested NaCl and KCl concentrations, respectively. Compared with the results obtained with no added salt, *Sa*BADH exhibited a higher activity at both 500 m*M* NaCl and 500 m*M* KCl, at which the enzyme has an average *R*_h_ of 5.55 ± 0.50 nm (molecular mass of 185.6 ± 0.7 kDa and PdI of 0.17 ± 0.07) and an average *R*_h_ of 5.05 ± 0.31 nm (molecular mass of 148.8 ± 0.2 kDa and PdI of 0.10 ± 0.06), respectively. Thus, the SEC-MALS and DLS data are consistent with the crystal structure, showing that the enzyme is predominantly tetrameric in the presence of NaCl, while more of the *Sa*BADH is dimeric in the presence of KCl.

### 3.4. The NAD-binding mode in *Sa*BADH and G234S-*Sa*BADH   

**Figure 5:**
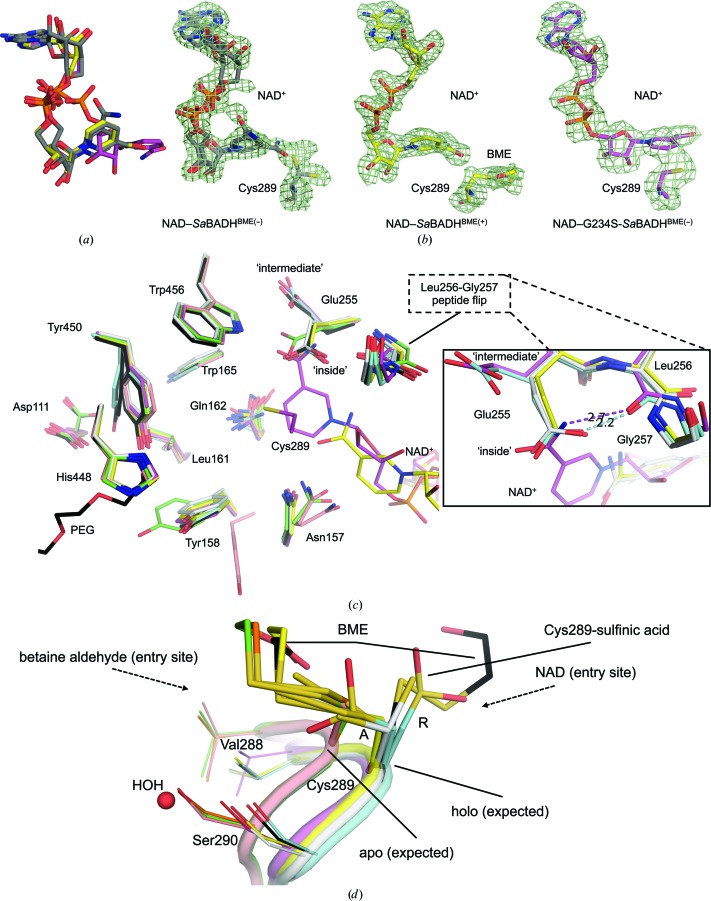
The NAD-binding site. (*a*) The ‘hydrolysis’ [NAD–*Sa*BADH^BME(+/−)^; C atoms in yellow/gray] and ‘hydride-transfer’ [NAD–G234S-*Sa*BADH^BME(−)^; C atoms in magenta] positions of NAD^+^. (*b*) The 3.0σ OMIT map (green mesh) of NAD^+^ in the NAD–(G234S-)*Sa*BADH structures [chains *A* are displayed and colored as in (*a*)]. (*c*) The catalytic and nicotinamide ring-binding sites in the *Sa*BADH structures: apo *Sa*BADH^BME(+)PEG^ (C atoms in black), apo *Sa*BADH^BME(+)^ (C atoms in green), apo *Sa*BADH^BME(−)^ (C atoms in pink), NAD–*Sa*BADH^BME(+)^ (C atoms in yellow), NAD–*Sa*BADH^BME(−)^ (C atoms in light gray), NAD–G234S-*Sa*BADH^BME(−)^ (C atoms in magenta), apo G234S-*Sa*BADH^BME(−)^ (C atoms in cyan) and apo G234S-*Sa*BADH^BME(+)^ (C atoms in orange). Cys289 and NAD^+^ in NAD–G234S-*Sa*BADH^BME(−)^ and NAD^+^ in NAD–*Sa*BADH^BME(+)^ are only shown for clarity. ‘Intermediate’ and ‘inside’ refer to the corresponding positions of the catalytic base Glu255. The bound PEG molecule at the NAD-binding site is not shown for clarity. The inset shows alternative conformations of the Leu256-Gly257 peptide bond and its possible role in supporting the ‘inside’ conformation of Glu255 and the ‘hydride-transfer’ position of NAD^+^; selected structures are shown and atoms are colored as in the main panel. (*d*) Attacking (A) and resting (R) conformations of Cys289 within all *Sa*BADH structures [chains *A* are displayed; C atoms are colored as in (*c*)].

NAD^+^ makes similar interactions with *Sa*BADH and G234S-*Sa*BADH (Supplementary Figs. S1*a*, S1*b* and S1*c*); *PROPKA* (Bas *et al.*, 2008 ▶; Li *et al.*, 2005 ▶) was used to estimate the p*K*_a_ values of the residues within the NAD-binding and substrate-binding sites in the presence/absence of NAD^+^ (Supplementary Table S1). A positively charged surface of the NAD-binding site accommodates the adenosine monophos­phate (AMP) moiety of the cofactor (Fig. 4 ▶ *d*). The adenine ring is positioned in a cleft between helix αF and helix αG (residues Gly236–Ile241), while the ribose of AMP and the pyrophosphate group are solvent-oriented (Fig. 4 ▶ *a*). The nicotinamide nucleotide (NMN) moiety is turned towards a negatively charged pocket in which the catalytic Cys289 is located (Figs. 4 ▶ *a* and 4 ▶ *d*). *Sa*BADH^BME(+/−)^ binds NAD^+^ in an orientation similar to the ‘hydrolysis’ conformation (Muñoz-Clares *et al.*, 2010 ▶) of the cofactor. Two alternative NAD^+^ conformations that differ in the orientation of the nicotinamide ring were modeled in NAD–*Sa*BADH^BME(−)^ to interpret the additional electron density (Figs. 5 ▶ *a* and 5 ▶ *b*). One of these NAD^+^ conformations aligns well with that in NAD–*Sa*BADH^BME(+)^. In NAD–G234S-*Sa*BADH^BME(−)^, the dinucleotide is in the ‘hydride-transfer’ position (Muñoz-Clares *et al.*, 2010 ▶), in which the amide group of NMN displaces the γ-carboxyl group of the catalytic base Glu255 towards its single ‘intermediate’ conformation. The C4N atom of the cofactor is ∼2.5 Å from the S^γ^ atom of the ‘attacking’ rotamer (González-Segura *et al.*, 2009 ▶) of Cys289 (Fig. 5 ▶ *c*). The wild-type apo and holo *Sa*BADH structures had Glu255 in the ‘inside’ and/or ‘intermediate’ but not the ‘outside’ conformations [as, for example, observed in human ALDH2; PDB entries 1zum (Larson *et al.*, 2005 ▶) and 2onp (Larson *et al.*, 2007 ▶)]. Structural comparison of all *Sa*BADH structures revealed that the Leu256-Gly257 peptide bond may undergo a conformational switch to secure the ‘hydride-transfer’ position of NAD^+^ or support the ‘inside’ position (González-Segura *et al.*, 2009 ▶) of Glu255 (Fig. 5 ▶ *c*).

### 3.5. Substrate-binding site of *Sa*BADH and G234S-*Sa*BADH   

The catalytic nucleophile Cys289 and the catalytic base Glu255 of *Sa*BADH were identified based on multiple sequence alignment with other BADHs. In all *Sa*BADH structures the side chain of Cys289 adopts two alternative conformations denoted ‘resting’ and ‘attacking’ (González-Segura *et al.*, 2009 ▶), but NAD–G234S-*Sa*BADH^BME(−)^, apo G234S-*Sa*BADH^BME(+)^ and NAD–*Sa*BADH^BME(+)^ only have the ‘attacking’ rotamer (Fig. 5 ▶ *d*). All (G234S-)*Sa*BADH^BME(+)^ structures have the ‘attacking’ conformer oxidized with BME, while both Cys289 conformers of apo *Sa*BADH^BME(+)PEG^ are BME-modified. Bulky electron density at the S^γ^ atom in NAD–*Sa*BADH^BME(+)^ (chains *B*, *C*, *E*, *G* and *H*) was interpreted as two alternative conformations of the BME–Cys289 adduct. The hydroxyl group of BME interacts with the Asn157 side chain and/or a water molecule. Both Cys289 rotamers in apo G234S-*Sa*BADH^BME(−)^ were modeled as cysteine-*S*-dioxide to explain the outsized electron density at the S^γ^ atom (Fig. 5 ▶ *d*).

Superposition of all eight *Sa*BADH structures revealed additional residues close to Cys289, Glu255 and the NMN-binding and substrate-binding areas for which the conformations varied depending on whether (G234S-)*Sa*BADH was in the apo or holo form (Fig. 5 ▶ *c*). Noticeable side-chain motions of Tyr158 and Tyr450 in apo G234S-*Sa*BADH^BME(−)^, NAD–G234S-*Sa*BADH^BME(−)^ and apo *Sa*BADH^BME(+)PEG^ (Fig. 5 ▶ *c*) may be functionally important for BA binding. Asn157 and Gln162 exhibited comparable smaller side-chain movements that may facilitate association/release of the cofactor/substrate/product (Fig. 5* ▶c* and Supplementary Fig. S1*b*).

### 3.6. Cation-binding sites in *Sa*BADH   

**Figure 6:**
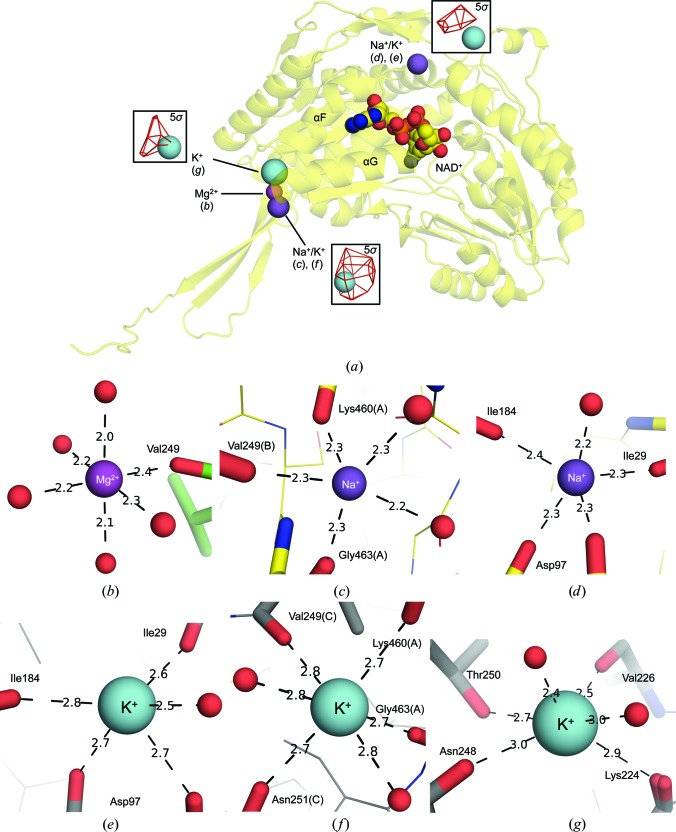
Binding of monovalent/divalent cations. (*a*) Localization of the metal-binding sites within *Sa*BADH (yellow ribbon). Positions of K^+^ were validated by anomalous difference Fourier maps (insets; red mesh; contoured at 5σ). NAD^+^ is shown in space-filling representation with atoms colored as follows: C, yellow; O, red; N, blue; P, orange. (*b*)–(*g*) Coordination geometries of the metal-binding sites within apo *Sa*BADH^BME(+)^ (*b*), NAD–*Sa*BADH^BME(+)^ (*c*, *d*) and NAD–*Sa*BADH^BME(−)^ (*e*, *f*, *g*). Water molecules are shown as red spheres.

Fig. 6 ▶ shows binding sites for Na^+^, K^+^ and Mg^2+^ at the dimerization interface of the protein and in the vicinity of the NAD-binding site. Binding of cations at similar sites have been reported previously (Muñoz-Clares *et al.*, 2010 ▶) and were shown to activate BADH/ALDH through facilitation of NAD(P)^+^ association and to affect the tertiary and quaternary structures of the enzymes (Falkenberg & Strøm, 1990 ▶; Valenzuela-Soto & Muñoz-Clares, 1994 ▶; Muñoz-Clares *et al.*, 2010 ▶; Velasco-García *et al.*, 1999 ▶; Valenzuela-Soto *et al.*, 2003 ▶; González-Segura *et al.*, 2013 ▶; Garza-Ramos *et al.*, 2013 ▶). Mg^2+^ (Fig. 6 ▶ *b*), Na^+^ (Fig. 6 ▶ *c*) and K^+^ (Fig. 6 ▶ *f*) bind at the dimerization interface and have one ligand in common, Val249; however, their modeled positions differ owing to their different coordination spheres. Alignment of the corresponding *Sa*BADH structures revealed that the aforementioned K^+^ and Na^+^ ions are just ∼0.8 Å apart, while Mg^2+^ is ∼2.4 and ∼2.7 Å from Na^+^ and K^+^, respectively. NAD–*Sa*BADH^BME(−)^ has an additional K^+^-binding site (Fig. 6 ▶ *g*) that is 6.1 Å away from that shown in Fig. 6 ▶(*f*). The modeled positions of Na^+^ (Fig. 6 ▶ *d*) and K^+^ (Fig. 6 ▶ *e*) are just ∼0.3 Å apart and are located near the NAD^+^-binding site between the NAD-binding domain and the catalytic domain.

## 4. Discussion   

### 4.1. The annotated *Sa*BADH is a functional BADH   

The putative *S. aureus* betaine aldehyde dehydrogenase enzyme SACOL2628 is indeed a functional BADH that utilizes NAD^+^ and BA as the primary cofactor and substrate, respectively (Fig. 1 ▶, Table 3 ▶). The activity is inhibited by high concentrations of BA. Kinetic data also show that *Sa*BADH is slightly more active in the presence of Na^+^ than in the presence of K^+^. DLS data suggest that Na^+^-induced tetramerization of *Sa*BADH may account for the observed higher activity compared with *Sa*BADH in the presence of K^+^. *Sa*BADH has maximal activity at 500 m*M* NaCl or KCl, and a further increase in the ionic strength may slightly reduce the activity by weakening the interactions required for NAD^+^ and BA binding without significantly affecting the oligomerization state.

NAD^+^ and NADH bind *Sa*BADH in a pH-dependent manner, with NADH having higher affinity for *Sa*BADH at pH 7.0 and 277 K (Fig. 2 ▶ *a*). Although the pH-dependence of NAD^+^ binding was not tested in SPR, *Sa*BADH activity and its co-crystallization with NAD^+^ at pH 8.0 suggest that the oxidized form of the cofactor binds more strongly at the alkaline pH. As suggested by our previously published mutagenesis (Chen *et al.*, 2014 ▶) and shown by the crystallo­graphic data presented here, initial NAD^+^ binding causes structural changes within the substrate-binding pocket that facilitate BA binding. Thus, the weak binding of BA to immobilized apo *Sa*BADH (Fig. 2 ▶ *d*) may be explained by the lack of these structural changes. BME modification of Cys289 may further affect the affinity of BA for the enzyme (right inset in Fig. 4 ▶ *a*, and Figs. 5 ▶ *c* and 5 ▶ *d*). Overall, we showed that immobilized *Sa*BADH remained stable and binds its cofactor and substrate over 2 d of SPR analysis. The empirically derived *K*_d_ values, however, need further careful re-quantification, since *Sa*BADH was tested in the presence of BME and DTT, which may affect the binding of NAD^+^/NADH and BA.

### 4.2. Structural–functional link between the NAD-binding and substrate-binding sites   

The highly conserved residues of the cofactor-binding site govern the NAD^+^–*Sa*BADH interactions similarly to other BADH/ALDHs (Figs. 4 ▶ *a* and 7 ▶ *a* and Supplementary Fig. S1). Comprehensive structural analysis of *Sa*BADH elaborates on how NAD^+^ binding traps the enzyme in a conformational state that facilitates the catalysis.

Firstly, the adenine ring of NAD^+^ imposes structural changes on helix αF that were also observed in apo G234S-*Sa*BADH^BME(+/−)^ and apo *Sa*BADH^BME(+)PEG^. Difference electron-density peaks around the Ala212–Glu215 segment of the helix of these apo structures suggest that this region might be flexible in general and that NAD^+^ binding selects or induces the favorable conformational state. The atoms of residues Ala212–Glu215 exhibit higher *B* factors than those of the adjacent residues.

**Figure 7:**
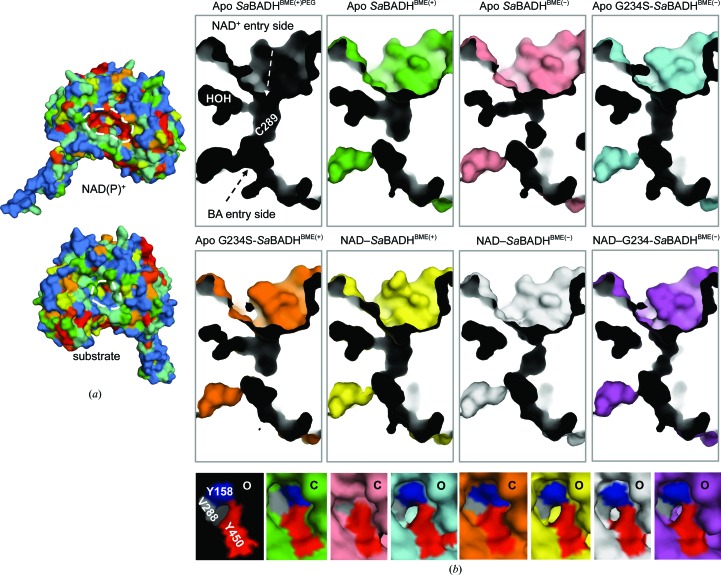

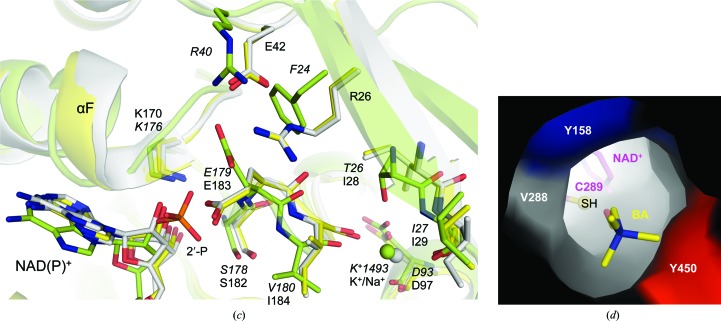
BADH enzymes. (*a*) Conservation of residues among *Sa*BADH (UniProtKB entry Q5HCU0), the human mitochondrial ALDH (UniProtKB entry P05091; PDB entry 1cw3; Ni *et al.*, 1999 ▶), *Ec*BADH (UniProtKB entry P77674; PDB entry 1wnb; Gruez *et al.*, 2004 ▶), *Pa*BADH (UniProtKB entry Q9HTJ1; PDB entry 2wme; González-Segura *et al.*, 2009 ▶), *So*BADH (UniProtKB entry P17202; PDB entry 4a0m; Díaz-Sánchez *et al.*, 2012 ▶), *At*BADH (UniProtKB entry Q8UH56; PDB entry 3r31; New York Structural Genomics Research Consortium, unpublished work) and *Gm*BADH (UniProtKB entry P56533; PDB entry 1bpw; Johansson *et al.*, 1998 ▶). Residues are colored from blue (variable) to red (100% conserved) and the *Sa*BADH structure is displayed. (*b*) NAD^+^ binding imposes structural changes on the substrate-binding site, creating a solvent-accessible channel between the NAD-binding and substrate-binding sites [surface representations (the C atoms) colored as in Fig. 5 ▶ *c*]. The large insets show whether the channel is open or closed. A solvent-accessible pocket (white ‘HOH’; composed of residues Gly257–Pro261, Gly281–Arg294 and Ile386–Thr397) may serve as a ‘buffer’ for the additional structural changes required for substrate binding. The small insets show whether the channel is open (O) or closed (C) at the substrate-binding site. Val288 (gray), Tyr158 (blue) and Tyr450 (red) may determine the size of the opening, which depends on the presence/absence of NAD^+^ and/or a small molecule, *i.e.* a substrate. (*c*) Local structural features/changes that may favor binding of the 2′-P group of NADP^+^ in *Pa*BADH (light green) and impede binding in *Sa*BADH [NAD–*Sa*BADH^BME(+)^, yellow; NAD–*Sa*BADH^BME(−)^, light gray]. The presence of Ile28 (*Sa*BADH) instead of the equivalent Thr26 (*Pa*BADH) may change the coordination geometry of a metal (shown as spheres; K^+^1493, light green, *Pa*BADH; Na^+^/K^+^, light gray, *Sa*BADH; see Figs. 6 ▶ *d* and 6 ▶ *e*) and thus possibly affect the lower affinity of *Sa*BADH for NADP^+^. (*d*) Merged model based on multiple structural superposition showing NAD^+^ and Cys289 from NAD–G234S-*Sa*BADH^BME(−)^ and residues Val288 (gray), Try158 (blue) and Tyr450 (red) from apo *Sa*BADH^BME(+)PEG^ and betaine aldehyde (BA; carbon, yellow; nitrogen, blue; oxygen, red) from human mitochondrial ALDH.

Secondly, with NAD^+^ in either the ‘hydrolysis’ or the ‘hydride-transfer’ position, the Val288–Ser290 loop that bears the catalytic Cys289 is displaced from its apo position (Figs. 5 ▶ *a* and 5 ▶ *c*). As a result of this relocation, an internal cavity around Cys289 expands and can be utilized for proper positioning of BA (Fig. 7 ▶ *b*). Oxidation of Cys289 to a sulfinic acid in apo G234S-*Sa*BADH^BME(−)^ and chemical mimicry of the PEG molecules in apo *Sa*BADH^BME(+)PEG^ at the NAD-binding and substrate-binding sites may prompt the loop to retreat (Figs. 4 ▶ *b* and 5 ▶ *d*). The Val288–Ser290 loop in apo G234S-*Sa*BADH^BME(−)^ and all NAD-bound *Sa*BADH structures is structurally aligned with an equivalent Asp279–Thr281 loop in the apo/holo structures of the *E. coli* BADH YdcW (PDB entries 1wnd and 1wnb; Gruez *et al.*, 2004 ▶). YdcW contains Ser225 instead of Gly234 (*Sa*BADH) and its catalytic Cys280 is reduced. Thus, the G234S substitution in *Sa*BADH may also contribute to the repositioning of the loop. A possible effect of the BME–Cys289 adduct on the loop repositioning in apo *Sa*BADH^BME(+)PEG^ is excluded because the loop is in the apo conformation in apo *Sa*BADH^BME(+)^.

Thirdly, the observed main-chain/side-chain flexibility of the active-site residues Asn157, Gln162, Glu255, Leu256 and Gly257 might facilitate cofactor and substrate binding (Fig. 5 ▶ *c* and Supplementary Fig. S1).

Fourthly, the NAD-dependent repositioning of the Val288–Ser290 loop may also cause changes in the main-chain/side-chain torsional angles of Asp111, Tyr158, His448 and Tyr450 located near the substrate-binding site (Figs. 5 ▶ *c* and 7 ▶ *a*). Based on our structural analysis, we propose that Asp111 and His448 may assist in BA binding by, for example, stabilizing the side chains of Tyr158 and Tyr450, which would in turn precisely guide/position BA in the vicinity of Cys289. Although the two tyrosine residues showed modest dihedral angle variations in the presence of NAD^+^ and substrate-mimicking molecules (Figs. 5 ▶ *c* and 5 ▶ *d*), their rotational freedom may still be limited by the neighboring Pro159, Trp165, Pro449 and Trp456. Further, intermolecular interactions at the tetramerization interface could reduce the conformational freedom of the loop (residues Ile444–Tyr459) that contains Tyr450.

Thus, upon NAD^+^ binding structural changes at the cofactor-docking site are further transmitted to the BA-binding area, facilitating recognition and binding of the substrate within a distance suitable for nucleophilic attack by Cys289.

### 4.3. The NAD-binding mode in *Sa*BADH   

The BME–Cys289 adduct and the G234S mutation determine the NAD-binding mode in *Sa*BADH. The adduct may mimic the intermediate enzyme–substrate complex and thus promote NAD^+^ binding in the ‘hydrolysis’ position in NAD–*Sa*BADH^BME(+)^. With the reduced Cys289, the two ‘hydrolysis’ positions were modeled in NAD–*Sa*BADH^BME(−)^, yet ambiguous electron density at the NAD-binding site may also indicate a mixture of the ‘hydrolysis’ and ‘hydride-transfer’ positions (Fig. 5 ▶ *b*). Traces of aldehydes in PEG-based crystallization conditions have been proposed to account for the dehydrogenase activity of human ALDH (PDB entry 1o00; Perez-Miller & Hurley, 2003 ▶). Since *Sa*BADH^BME(−)^ was purified and co-crystallized with NAD^+^ from PEG at a pH of ∼8.0, at which it exhibited maximum activity (Fig. 1 ▶ *a*), it is possible that *Sa*BADH^BME(−)^ was turning over in the crystal. The lack of a serine residue at position 234, which stabilizes the ‘hydride-transfer’ position of NAD^+^ in NAD–G234S-*Sa*BADH^BME(−)^, may also account for the observed flexibility of NAD^+^ in NAD–*Sa*BADH^BME(−)^. When the NAD–G234S-*Sa*BADH^BME(−)^ and NAD–*Sa*BADH^BME(−)^ structures were superimposed, the NAD^+^ ‘hydride-transfer’ conformer of the former structure fitted the aforementioned uncertain electron density of the latter structure. An alternative interpretation is that NAD^+^ and *Sa*BADH^BME(−)^ may form a covalent adduct similar to that between NADP^+^ and *Pa*BADH (PDB entry 2wox; Díaz-Sánchez *et al.*, 2011 ▶). However, such a covalent adduct could not explain the observed NAD^+^ electron-density peaks. Furthermore, the conformations of Cys289 and Glu255 of NAD–*Sa*BADH^BME(−)^ would not allow dinucleotide–Cys adduct formation. Thus, BME oxidation of Cys289 and the G234S mutation helped to trap the ‘hydrolysis’ and ‘hydride-transfer’ positions of the cofactor, respectively, allowing analysis of these binding modes in *Sa*BADH.

### 4.4. Preference for NAD^+^ over NADP^+^ in *Sa*BADH   

BADHs efficiently use NAD^+^/NADP^+^ (Mori *et al.*, 1992 ▶; Velasco-García *et al.*, 2000 ▶), while *Sa*BADH prefers NAD^+^ (Table 3 ▶). Comparison of the NADP–*Pa*BADH (PDB entry 2wme; González-Segura *et al.*, 2009 ▶) and NAD–*Sa*BADH structures suggested a rationale for preferential NAD^+^ binding in *Sa*BADH. In NADP–*Pa*BADH, Lys176, Ser178, Glu179, Gly207 and Gly209 (Lys180, Ser182, Glu183, Gly211 and Gly213 in *Sa*BADH) coordinate the 2′-phosphate group (2′-P) of NADP^+^ (Fig. 7 ▶ *c* and Supplementary Fig. S1*d*). The 2′-P displaces the side chain of Glu179 (Glu183 in *Sa*BADH) and makes a salt bridge with Arg40 (Glu42 in *Sa*BADH). Similar contacts and rearrangements within *Sa*BADH would be expected in the presence of NADP^+^. However, sequence variations at the 2′-P docking area and metal-binding site ∼11 Å away from the 2′-P binding area may selectively favor the binding of one form of the cofactor over another. K^+^ (K^+^1493 in *Pa*BADH) and Na^+^/K^+^ (*Sa*BADH) (Figs. 6 ▶ *d*, 6 ▶ *e* and 7 ▶ *c*) occupy the referred metal-binding site, yet their coordination geometries differ in the two structures. The hydroxyl group of Thr26 of *Pa*BADH (Ile28 in *Sa*BADH) binds K^+^1493, while a water molecule substitutes for the OH group of Thr26 in *Sa*BADH. Differences in the metal-coordination spheres seem to influence the position of the Ser178–Val180 loop of *Pa*BADH (residues Ser182–Ile184 in *Sa*BADH), which is closer to the 2′-P binding site in *Pa*BADH than in *Sa*BADH (Fig. 7 ▶ *b* and Supplementary Fig. S1*d*), hence making the 2′-P–*Pa*BADH interactions stronger. Based on this observation, the weaker affinity of *Sa*BADH for NADP^+^ may then be a consequence of the Thr26-to-Ile28 substitution and sequence variations within the 2′-P binding pocket.

## 5. Conclusions   

The high osmolarity burden in *S. aureus* can be handled by (i) the accumulation of the exogenous osmolyte betaine and (ii) its biosynthesis from choline. The second pathway generates a toxic BA intermediate that is oxidized by BADH to betaine. Increasing experimental evidence suggests an important role for BADHs in the survival of pathogenic bacteria, which in turn emphasizes the need for a detailed evaluation of the structure and function of the enzymes. We studied a putative BADH from an early MRSA COL isolate, denoted *Sa*BADH, and showed that it is a functional NAD^+^-dependent BADH that utilizes BA as the primary substrate. The affinity of *Sa*BADH for NAD^+^/NADH/BA depends on pH, temperature and buffer composition, although it was also affected by BME/DTT oxidation of the catalytic Cys289. The enzymatic activity is quite tolerant to high ionic strength, yet may be attenuated at high ionic strength. The solution data are supported by the structural analysis and emphasize the importance of the NAD^+^-driven structural rearrangements at the cofactor-binding and substrate-binding sites for enzymatic activity. The Val288–Ser290 loop that contains the catalytic Cys289 repositions in the presence of NAD^+^, creating room for the nucleophilic reaction. While NAD^+^ is bound, Tyr158 and Try450 at the substrate-binding site may play the role of active-site ‘gatekeepers’ and help to position the substrate at a distance suitable for nucleophilic attack. Overall, extensive structural analysis and preliminary ligand-binding data support our previously published comprehensive structure-based mutagenesis data on the ordered bi-bi kinetic mechanism, substrate inhibition and existence of a structural–functional link between the NAD-binding and substrate-binding sites (Chen *et al.*, 2014 ▶).

## Supplementary Material

PDB reference: betaine aldehyde dehydrogenase, 4mpb

PDB reference: complex with NAD^+^, 4mpy

PDB reference: complex with NAD^+^ with BME-free Cys289, 4nea

PDB reference: with BME-free Cys289, 4nu9

PDB reference: with BME-modified Cys289 and PEG molecule in active site, 4qto

PDB reference: G234S mutant, in complex with NAD^+^ with BME-free Cys289, 4qn2

PDB reference: G234S mutant, with BME-modified Cys289, 4q92

PDB reference: G234S mutant, with BME-free sulfinic acid form of Cys289, 4qje

Supporting Information.. DOI: 10.1107/S1399004715004228/mn5083sup2.pdf

## Figures and Tables

**Table 1:** Data-collection and refinement statistics for the wild-type *Sa*BADH structures Values in parentheses are for the highest resolution shell.

	Apo *Sa*BADH^BME(+)^	NAD*Sa*BADH^BME(+)^	Apo *Sa*BADH^BME()^	NAD*Sa*BADH^BME()^	Apo *Sa*BADH^BME(+)PEG^
PDB entry	4mpb	4mpy	4nu9	4nea	4qto
Data collection					
Wavelength ()	0.97872	0.9184	0.97872	0.97856	0.97856
Data-collection temperature (K)	100	100	100	100	100
Space group	*P*2_1_2_1_2	*P*2_1_	*P*2_1_2_1_2	*P*2_1_2_1_2_1_	*C*2
Unit-cell parameters					
*a* ()	106.3	99.8	106.6	86.7	224.3
*b* ()	118.6	159.1	118.4	142.1	102.5
*c* ()	87.9	122.9	88.5	163.1	118.2
()	90.0	90.0	90.0	90.0	90.0
()	90.0	94.8	90.0	90.0	104.5
()	90.0	90.0	90.0	90.0	90.0
Resolution range ()	88.051.70 (1.761.70)	25.001.85 (1.881.85)	30.002.30 (2.342.30)	30.001.90 (1.931.90)	30.001.65 (1.681.65)
No. of reflections	122661 (12121)	324147 (16115)	48647 (2474)	155760 (7884)	307248 (15078)
*R*_merge_ (%)	7.4 (52.6)	9.6 (43.1)	10.7 (50.1)	8.2 (44.6)	8.2 (63.1)
Completeness (%)	100.0 (100.0)	100.0 (100.0)	96.9 (100.0)	97.9 (100.0)	98.6 (97.5)
*I*/**(*I*)	19.9 (2.5)	12.8 (3.2)	18.4 (4.4)	22.7 (4.6)	16.7 (2.5)
Multiplicity	5.9 (4.2)	4.1 (4.0)	7.3 (7.5)	7.2 (7.2)	4.3 (4.3)
Wilson *B* factor (^2^)	27.7	25.6	30.4	24.4	17.0
Refinement					
Resolution range ()	19.791.70 (1.741.70)	24.981.85 (1.891.85)	29.872.30 (2.342.30)	29.851.90 (1.951.90)	29.711.65 (1691.65)
No. of reflections	116223 (8449)	314222 (22471)	46132 (3244)	147578 (7781)	290362 (21045)
*R*_work_/*R*_free_ (%)	13.8/17.5 (25.5/28.9)	12.5/16.6 (17.0/21.7)	15.9/21.5 (19.4/25.8)	13.9/18.8 (19.2/23.0)	13.6/15.6 (21.0/24.5)
Protein molecules	2	8	2	4	4
Protein atoms	8505	32200	7807	16488	16509
Solvent atoms	1385	4722	610	1996	2742
Heterogen atoms	6	371	4	368	129
Mean temperature factor (^2^)					
Overall	23.9	19.9	25.6	27.2	19.7
Protein	21.9	18.5	25.2	26.0	17.3
Solvent atoms	35.8	30.1	30.7	36.3	32.8
Heterogen atoms	41.3	20.3	25.4	31.7	47.9
Coordinate deviation					
R.m.s.d., bonds ()	0.014	0.013	0.014	0.014	0.011
R.m.s.d., angles ()	1.694	1.722	1.705	1.698	1.643
Ramachandran plot†					
Most favored (%)	92.9	93.5	92.1	93.2	93.6
Allowed (%)	6.3	5.9	7.1	6.2	5.7
Generously allowed (%)	0.6	0.5	0.5	0.3	0.5
Disallowed (%)	0.2	0.1	0.3	0.3	0.2

† Statistics are based on *PROCHECK* (Laskowski *et al.*, 1993 ▶).

**Table 2:** Data-collection and refinement statistics for the G234S-*Sa*BADH mutant structures Values in parentheses are for the highest resolution shell.

	Apo G234S-*Sa*BADH^BME()^	Apo G234S-*Sa*BADH^BME(+)^	NADG234S-*Sa*BADH^BME()^
PDB entry	4qje	4q92	4qn2
Data collection			
Wavelength ()	0.97872	0.97872	0.97872
Data-collection temperature (K)	100	100	100
Space group	*C*2	*C*2	*P*2_1_
Unit-cell parameters			
*a* ()	218.7	157.4	88.6
*b* ()	102.9	156.4	168.4
*c* ()	118.1	88.4	144.5
()	101.4	110.0	104.9
Resolution range ()	30.001.85 (1.881.85)	30.001.90 (1.931.90)	30.002.60 (2.642.60)
No. of reflections	217702 (10794)	155391 (7680)	117959 (6068)
*R*_merge_ (%)	10.0 (63.4)	10.4 (58.5)	10.4 (60.7)
Completeness (%)	99.9 (99.6)	98.8 (97.9)	93.7 (97.1)
*I*/**(*I*)	15.9 (2.3)	17.43 (2.4)	12.8 (2.34)
Multiplicity	3.8 (3.6)	3.9 (3.9)	3.9 (3.8)
Wilson *B* factor (^2^)	17.6	21.0	51.9
Refinement			
Resolution range ()	29.831.85 (1.891.85)	29.501.90 (1.951.90)	29.782.60 (2.602.67)
No. of reflections	206767 (14864)	147596 (10678)	111581 (8480)
*R*_work_/*R*_free_ (%)	14.9/17.5 (23.8/26.6)	19.3/22.3 (25.8/30.5)	18.4/21.9 (27.0/31.4)
Protein molecules	4	4	8
Protein atoms	16348	16069	30871
Solvent atoms	2511	1614	707
Heterogen atoms	/126	20	356
Mean temperature factor (^2^)			
Overall	21.9	25.1	42.4
Protein	20.1	24.7	42.7
Solvent atoms	32.9	28.8	34.5
Heterogen atoms	46.4	28.6	38.4
Coordinate deviation			
R.m.s.d., bonds ()	0.010	0.012	0.012
R.m.s.d., angles ()	1.537	1.513	1.587
Ramachandran plot†			
Most favored (%)	92.7	93.7	91.6
Allowed (%)	6.5	5.5	7.7
Generously allowed (%)	0.6	0.5	0.4
Disallowed (%)	0.2	0.2	0.3

† Statistics are based on *PROCHECK* (Laskowski *et al.*, 1993 ▶).

**Table 3:** *Sa*BADH kinetic parameters

	NAD^+^	NADP^+^	Betaine aldehyde
*K*_m_ (m*M*)	0.43 0.03	2.26 0.35	0.17 0.03
*K*_i_ (m*M*)	N/A	N/A	0.34 0.06
*V*_max_ (molmin^1^mg^1^)	6.78 0.16	0.66 0.08	12.1 1.5
*k*_cat_ (s^1^)	6.18 0.14	0.56 0.07	11.0 1.4

## References

[bb1] Abboud, F. M. & Waisbren, B. A. (1959). *AMA Arch. Intern. Med.* **104**, 226–233. 10.1001/archinte.1959.00270080052006 13669776

[bb3] Archer, D. L. (1996). *Trends Food Sci. Technol.* **7**, 91–95.

[bb4] Armand-Lefevre, L., Ruimy, R. & Andremont, A. (2005). *Emerg. Infect. Dis.* **11**, 711–714. 10.3201/eid1105.040866 PMC3320358 15890125

[bb5] Bae, J.-H., Anderson, S. H. & Miller, K. J. (1993). *Appl. Environ. Microbiol.* **59**, 2734–2736. 10.1128/aem.59.8.2734-2736.1993 PMC182349 8368857

[bb6] Bas, D. C., Rogers, D. M. & Jensen, J. H. (2008). *Proteins*, **73**, 765–783. 10.1002/prot.22102 18498103

[bb7] Boch, J., Kempf, B., Schmid, R. & Bremer, E. (1996). *J. Bacteriol.* **178**, 5121–5129. 10.1128/jb.178.17.5121-5129.1996 PMC178307 8752328

[bb8] Boch, J., Nau-Wagner, G., Kneip, S. & Bremer, E. (1997). *Arch. Microbiol.* **168**, 282–289. 10.1007/s002030050500 9297465

[bb9] Cánovas, D., Vargas, C., Kneip, S., Morón, M. J., Ventosa, A., Bremer, E. & Nieto, J. J. (2000). *Microbiology*, **146**, 455–463. 10.1099/00221287-146-2-455 10708384

[bb10] Chambers, H. F. & Hackbarth, C. J. (1987). *Antimicrob. Agents Chemother.* **31**, 1982–1988. 10.1128/aac.31.12.1982 PMC175839 3439805

[bb11] Chan, P. F. & Foster, S. J. (1998). *J. Bacteriol.* **180**, 6232–6241. 10.1128/jb.180.23.6232-6241.1998 PMC107708 9829932

[bb12] Chen, V. B., Arendall, W. B., Headd, J. J., Keedy, D. A., Immormino, R. M., Kapral, G. J., Murray, L. W., Richardson, J. S. & Richardson, D. C. (2010). *Acta Cryst.* D**66**, 12–21. 10.1107/S0907444909042073 PMC2803126 20057044

[bb13] Chen, C., Joo, J. C., Brown, G., Stolnikova, E., Halavaty, A. S., Savchenko, A., Anderson, W. F. & Yakunin, A. F. (2014). *Appl. Environ. Microbiol.* **80**, 3992–4002. 10.1128/AEM.00215-14 PMC4054205 24747910

[bb14] Cosgrove, S. E., Sakoulas, G., Perencevich, E. N., Schwaber, M. J., Karchmer, A. W. & Carmeli, Y. (2006). *Clin. Infect. Dis.* **36**, 53–59. 10.1086/345476 12491202

[bb15] Craig, S. A. S. (2004). *Am. J. Clin. Nutr.* **80**, 539–549. 10.1093/ajcn/80.3.539 15321791

[bb16] Cuny, C., Friedrich, A., Kozytska, S., Layer, F., Nübel, U., Ohlsen, K., Strommenger, B., Walther, B., Wieler, L. & Witte, W. (2010). *Int. J. Med. Microbiol.* **300**, 109–117. 10.1016/j.ijmm.2009.11.002 20005777

[bb17] Davis, I. W., Leaver-Fay, A., Chen, V. B., Block, J. N., Kapral, G. J., Wang, X., Murray, L. W., Arendall, W. B., Snoeyink, J., Richardson, J. S. & Richardson, D. C. (2007). *Nucleic Acids Res.* **35**, W375–383. 10.1093/nar/gkm216 PMC1933162 17452350

[bb18] DeLeo, F. R. & Chambers, H. F. (2009). *J. Clin. Invest.* **119**, 2464–2474. 10.1172/JCI38226 PMC2735934 19729844

[bb19] Díaz-Sánchez, Á. G., González-Segura, L., Mújica-Jiménez, C., Rudiño-Piñera, E., Montiel, C., Martínez-Castilla, L. P. & Muñoz-Clares, R. A. (2012). *Plant Physiol.* **158**, 1570–1582. 10.1104/pp.112.194514 PMC3343730 22345508

[bb20] Díaz-Sánchez, Á. G., González-Segura, L., Rudiño-Piñera, E., Lira-Rocha, A., Torres-Larios, A. & Muñoz-Clares, R. A. (2011). *Biochem. J.* **439**, 443–452. 10.1042/BJ20110376 21732915

[bb23] Eady, E. A. & Cove, J. H. (2003). *Curr. Opin. Infect. Dis.* **16**, 103–124. 10.1097/00001432-200304000-00007 12734443

[bb24] Emsley, P. & Cowtan, K. (2004). *Acta Cryst.* D**60**, 2126–2132. 10.1107/S0907444904019158 15572765

[bb25] Emsley, P., Lohkamp, B., Scott, W. G. & Cowtan, K. (2010). *Acta Cryst.* D**66**, 486–501. 10.1107/S0907444910007493 PMC2852313 20383002

[bb26] Eriksen, N. H. R., Espersen, F., Rosdahl, V. T. & Jensen, K. (1995). *Epidemiol. Infect.* **115**, 51–60. 10.1017/s0950268800058118 PMC2271555 7641838

[bb27] Falkenberg, P. & Strøm, A. R. (1990). *Biochim. Biophys. Acta*, **1034**, 253–259. 10.1016/0304-4165(90)90046-y 2194570

[bb28] Feldman, R. I. & Weiner, H. (1972). *J. Biol. Chem.* **247**, 267–272. 4336042

[bb29] Fitzgerald, T. L., Waters, D. L. E. & Henry, R. J. (2009). *Plant Biol.* **11**, 119–130. 10.1111/j.1438-8677.2008.00161.x 19228319

[bb30] Fluit, A. C. (2012). *Clin. Microbiol. Infect.* **18**, 735–744. 10.1111/j.1469-0691.2012.03846.x 22512702

[bb31] Gadda, G. & McAllister-Wilkins, E. E. (2003). *Appl. Environ. Microbiol.* **69**, 2126–2132. 10.1128/AEM.69.4.2126-2132.2003 PMC154813 12676692

[bb32] Garza-Ramos, G., Mújica-Jiménez, C. & Muñoz-Clares, R. A. (2013). *PLoS One*, **8**, e54899. 10.1371/journal.pone.0054899 PMC3554688 23365686

[bb33] Gill, S. R. *et al.* (2005). *J. Bacteriol.* **187**, 2426–2438. 10.1128/JB.187.7.2426-2438.2005 PMC1065214 15774886

[bb34] González-Segura, L., Riveros-Rosas, H., Díaz-Sánchez, Á. G., Julián-Sánchez, A. & Muñoz-Clares, R. A. (2013). *Chem. Biol. Interact.* **202**, 41–50. 10.1016/j.cbi.2012.12.007 23295228

[bb35] González-Segura, L., Rudiño-Piñera, E., Muñoz-Clares, R. A. & Horjales, E. (2009). *J. Mol. Biol.* **385**, 542–557. 10.1016/j.jmb.2008.10.082 19013472

[bb36] Graham, J. E. & Wilkinson, B. J. (1992). *J. Bacteriol.* **174**, 2711–2716. 10.1128/jb.174.8.2711-2716.1992 PMC205912 1556089

[bb37] Gruez, A., Roig-Zamboni, W., Grisel, S., Salomoni, A., Valencia, C., Campanacci, V., Tegoni, M. & Cambillau, C. (2004). *J. Mol. Biol.* **343**, 29–41. 10.1016/j.jmb.2004.08.030 15381418

[bb38] Halavaty, A. S., Borek, D., Tyson, G. H., Veesenmeyer, J. L., Shuvalova, L., Minasov, G., Otwinowski, Z., Hauser, A. R. & Anderson, W. F. (2012). *PLoS One*, **7**, e49388. 10.1371/journal.pone.0049388 PMC3498133 23166655

[bb39] Holm, L. & Park, J. (2000). *Bioinformatics*, **16**, 566–567. 10.1093/bioinformatics/16.6.566 10980157

[bb40] Holm, L. & Rosenström, P. (2010). *Nucleic Acids Res.* **38**, W545–W549. 10.1093/nar/gkq366 PMC2896194 20457744

[bb41] Jackson, B., Brocker, C., Thompson, D. C., Black, W., Vasiliou, K., Nebert, D. W. & Vasiliou, V. (2011). *Hum. Genomics*, **5**, 283–303. 10.1186/1479-7364-5-4-283 PMC3392178 21712190

[bb42] Jevons, M. P. (1961). *BMJ*, **1**, 124–125.

[bb43] Johansson, K., Ramaswamy, S., Eklund, H., El-Ahmad, M., Hjelmqvist, L. & Jörnvall, H. (1998). *Protein Sci.* **7**, 2106–2117. 10.1002/pro.5560071007 PMC2143847 9792097

[bb44] Kaenjak, A., Graham, J. E. & Wilkinson, B. J. (1993). *J. Bacteriol.* **175**, 2400–2406. 10.1128/jb.175.8.2400-2406.1993 PMC204529 8468298

[bb45] Kapfhammer, D., Karatan, E., Pflughoeft, K. J. & Watnick, P. I. (2005). *Appl. Environ. Microbiol.* **71**, 3840–3847. 10.1128/AEM.71.7.3840-3847.2005 PMC1169069 16000796

[bb46] Katzif, S., Danavall, D., Bowers, S., Balthazar, J. T. & Shafer, W. M. (2003). *Infect. Immun.* **71**, 4304–4312. 10.1128/IAI.71.8.4304-4312.2003 PMC166043 12874306

[bb47] Kluytmans, J. A. (2010). *Clin. Microbiol. Infect.* **16**, 11–15.

[bb48] Kopečny, D., Končitíková, R., Tylichová, M., Vigouroux, A., Moskalíková, H., Soural, M., Šebela, M. & Moréra, S. (2013). *J. Biol. Chem.* **288**, 9491–9507. 10.1074/jbc.M112.443952 PMC3611018 23408433

[bb49] Krissinel, E. & Henrick, K. (2007). *J. Mol. Biol.* **372**, 774–797. 10.1016/j.jmb.2007.05.022 17681537

[bb50] Lamark, T., Kaasen, I., Eshoo, M. W., Falkenberg, P., McDougall, J. & Strøm, A. R. (1991). *Mol. Microbiol.* **5**, 1049–1064. 10.1111/j.1365-2958.1991.tb01877.x 1956285

[bb51] Larson, H. N., Weiner, H. & Hurley, T. D. (2005). *J. Biol. Chem.* **280**, 30550–30556. 10.1074/jbc.M502345200 PMC1262676 15983043

[bb52] Larson, H. N., Zhou, J., Chen, Z., Stamler, J. S., Weiner, H. & Hurley, T. D. (2007). *J. Biol. Chem.* **282**, 12940–12950. 10.1074/jbc.M607959200 PMC1885376 17327228

[bb53] Laskowski, R. A., MacArthur, M. W., Moss, D. S. & Thornton, J. M. (1993). *J. Appl. Cryst.* **26**, 283–291.

[bb54] Laskowski, R. A. & Swindells, M. B. (2011). *J. Chem. Inf. Model.* **51**, 2778–2786. 10.1021/ci200227u 21919503

[bb55] Li, H., Robertson, A. D. & Jensen, J. H. (2005). *Proteins*, **61**, 704–721. 10.1002/prot.20660 16231289

[bb56] Light, S. H., Anderson, W. F. & Lavie, A. (2013). *Protein Sci.* **22**, 418–424. 10.1002/pro.2218 PMC3610047 23341204

[bb57] Majorek, K. A., Kuhn, M. L., Chruszcz, M., Anderson, W. F. & Minor, W. (2014). *Protein Sci.* **23**, 1359–1368. 10.1002/pro.2520 PMC4286991 25044180

[bb58] Matthews, P. R. & Stewart, P. R. (1984). *FEMS Microbiool. Rev.* **22**, 161–166.

[bb59] McCoy, A. J., Grosse-Kunstleve, R. W., Adams, P. D., Winn, M. D., Storoni, L. C. & Read, R. J. (2007). *J. Appl. Cryst.* **40**, 658–674. 10.1107/S0021889807021206 PMC2483472 19461840

[bb60] McDowell, D. A. (2004). *Safety Assurance During Food Processing: Food Safety Assurance and Veterinary Public Health*, edited by F. J. M. Smulders & J. D. Collins, pp. 243–265. Wageningen: Wageningen Academic Publishers.

[bb61] McMahon, M. A., Xu, J., Moore, J. E., Blair, I. S. & McDowell, D. A. (2007). *Appl. Environ. Microbiol.* **73**, 211–217. 10.1128/AEM.00578-06 PMC1797128 17142359

[bb62] Mendum, M. L. & Smith, L. T. (2002). *Appl. Environ. Microbiol.* **68**, 813–819. 10.1128/AEM.68.2.813-819.2002 PMC126668 11823223

[bb63] Minor, W., Cymborowski, M., Otwinowski, Z. & Chruszcz, M. (2006). *Acta Cryst.* D**62**, 859–866. 10.1107/S0907444906019949 16855301

[bb64] Moellering, R. C. Jr (2012). *J. Antimicrob. Chemother.* **67**, 4–11. 10.1093/jac/dkr437 22010206

[bb65] Mori, N., Yoshida, N. & Kitamoto, Y. (1992). *J. Ferment. Bioeng.* **73**, 352–356.

[bb66] Morris, R. J., Perrakis, A. & Lamzin, V. S. (2003). *Methods Enzymol.* **374**, 229–244. 10.1016/S0076-6879(03)74011-7 14696376

[bb67] Muñoz-Clares, R. A., Díaz-Sánchez, Á. G., González-Segura, L. & Montiel, C. (2010). *Arch. Biochem. Biophys.* **493**, 71–81. 10.1016/j.abb.2009.09.006 19766587

[bb68] Murshudov, G. N., Skubák, P., Lebedev, A. A., Pannu, N. S., Steiner, R. A., Nicholls, R. A., Winn, M. D., Long, F. & Vagin, A. A. (2011). *Acta Cryst.* D**67**, 355–367. 10.1107/S0907444911001314 PMC3069751 21460454

[bb69] Ni, L., Zhou, J., Weiner, H. & Hurley, T. D. (1999). *Protein Sci.* **8**, 2784–2790. 10.1110/ps.8.12.2784 PMC2144226 10631996

[bb70] Otto, M. (2010). *Exp. Rev. Dermatol.* **5**, 183–195. 10.1586/edm.10.6 PMC2867359 20473345

[bb71] Otwinowski, Z. & Minor, W. (1997). *Methods Enzymol.* **276**, 307–326. 10.1016/S0076-6879(97)76066-X 27754618

[bb72] Painter, J. & Merritt, E. A. (2006). *Acta Cryst.* D**62**, 439–450. 10.1107/S0907444906005270 16552146

[bb73] Pantosti, A. (2012). *Front. Microbiol.* **3**, 127. 10.3389/fmicb.2012.00127 PMC3321498 22509176

[bb75] Paulsen, I. T., Firth, M. & Skurray, R. A. (1997). *The Staphylococci in Human Disease*, edited by K. B. Crossley & G. L. Archer, pp. 175–212. New York: Churchill Livingstone.

[bb76] Perez-Miller, S. J. & Hurley, T. D. (2003). *Biochemistry*, **42**, 7100–7109. 10.1021/bi034182w 12795606

[bb77] Rickard, A. H., Lindsay, S., Lockwood, G. B. & Gilbert, P. (2004). *J. Appl. Microbiol.* **97**, 1063–1068. 10.1111/j.1365-2672.2004.02401.x 15479423

[bb78] Roessler, M. & Muller, V. (2001). *Environ. Microbiol.* **3**, 743–754. 10.1046/j.1462-2920.2001.00252.x 11846768

[bb79] Rohrer, S., Maki, H. & Berger-Bächi, B. (2003). *J. Med. Microbiol.* **52**, 605–607. 10.1099/jmm.0.05176-0 12867551

[bb80] Rosenstein, R., Futter-Bryniok, D. & Götz, F. (1999). *J. Bacteriol.* **181**, 2273–2278. 10.1128/jb.181.7.2273-2278.1999 PMC93644 10094709

[bb81] Rowan, N. J. (1999). *Trends Food Sci. Technol.* **10**, 261–270.

[bb82] Scheffler, R. J., Colmer, S., Tynan, H., Demain, A. L. & Gullo, V. P. (2013). *Appl. Microbiol. Biotechnol.* **97**, 969–978. 10.1007/s00253-012-4609-8 23233204

[bb83] Sleator, R. D. & Hill, C. (2002). *FEMS Microbiool. Rev.* **26**, 49–71. 10.1111/j.1574-6976.2002.tb00598.x 12007642

[bb85] Tsai, M., Ohniwa, R. L., Kato, Y., Takeshita, S. L., Ohta, T., Saito, S., Hayashi, H. & Morikawa, K. (2011). *BMC Microbiol.* **11**, 13. 10.1186/1471-2180-11-13 PMC3030509 21241511

[bb86] Tylichová, M., Kopečný, D., Moréra, S., Briozzo, P., Lenobel, R., Snégaroff, J. & Šebela, M. (2010). *J. Mol. Biol.* **396**, 870–882. 10.1016/j.jmb.2009.12.015 20026072

[bb87] Ueland, P. M. (2011). *J. Inherit. Metab. Dis.* **34**, 3–15. 10.1007/s10545-010-9088-4 20446114

[bb88] Valenzuela-Soto, E. M. & Muñoz-Clares, R. A. (1994). *J. Plant Physiol.* **143**, 145–152.

[bb89] Valenzuela-Soto, E. M., Velasco-García, R., Mújica-Jiménez, C., Gaviria-González, L. & Muñoz-Clares, R. A. (2003). *Chem. Biol. Interact.* **143–144**, 139–148. 10.1016/s0009-2797(02)00198-9 12604198

[bb90] Velasco-García, R., González-Segura, L. & Muñoz-Clares, R. A. (2000). *Biochem. J.* **352**, 675–683. PMC1221504 11104673

[bb91] Velasco-García, R., Mújica-Jiménez, C., Mendoza-Hernández, G. & Muñoz-Clares, R. A. (1999). *J. Bacteriol.* **181**, 1292–1300. 10.1128/jb.181.4.1292-1300.1999 PMC93508 9973357

[bb92] Vijaranakul, U., Nadakavukaren, M. J., de Jonge, B. L., Wilkinson, B. J. & Jayaswal, R. K. (1995). *J. Bacteriol.* **177**, 5116–5121. 10.1128/jb.177.17.5116-5121.1995 PMC177291 7665491

[bb93] Walsh, C. (1999). *Science*, **284**, 442–443. 10.1126/science.284.5413.442 10232990

[bb94] Wargo, M. J. (2013). *PLoS One*, **8**, e56850. 10.1371/journal.pone.0056850 PMC3572970 23457628

[bb95] Wendlandt, S., Schwarz, S. & Silley, P. (2013). *Annu. Rev. Food. Sci. Technol.* **4**, 117–139. 10.1146/annurev-food-030212-182653 23190141

[bb96] Wenzel, R. P. (2004). *N. Engl. J. Med.* **351**, 523–526. 10.1056/NEJMp048093 15295041

[bb97] Winn, M. D. *et al.* (2011). *Acta Cryst.* D**67**, 235–242.

[bb98] Zheng, H., Chordia, M. D., Cooper, D. R., Chruszcz, M., Müller, P., Sheldrick, G. M. & Minor, W. (2014). *Nature Protoc.* **9**, 156–170. 10.1038/nprot.2013.172 PMC4410975 24356774

