# Supplementary material for: Structural and functional analysis of betaine aldehyde dehydrogenase from Staphylococcus aureus
Source: Acta Crystallogr D Biol Crystallogr. 2015 Apr 25;71(Pt 5):1159–75. doi: 10.1107/S1399004715004228 (PMC4427200; doi:10.1107/S1399004715004228)
Supplement: Supplementary file 1 [file d-71-01159-sup2.pdf]

BIOLOGICAL
CRYSTALLOGRAPHY

Volume 71 (2015)

Supporting information for article:

Structural and functional analysis of betaine aldehyde dehydrogenase from *Staphylococcus aureus*

Andrei S. Halavaty, Rebecca L. Rich, Chao Chen, Jeong Chan Joo, George Minasov, Ievgeniia Dubrovska, James R. Winsor, David G. Myszka, Mark Duban, Ludmilla Shuvalova, Alexander F. Yakunin and Wayne F. Anderson

Table S1. Changes in pKa values of some residues of the NAD- and substrate-binding sites.

	Wild-type					G234S mutant			
	4mpb	4mpy	4nu9	4nea	4qto	4qje	4q92	4qn2	
		NAD^+		NAD^+				NAD^+	
Residue[a]									model pKa
Asp111	7.44	6.83	7.02	8.49	6.76	6.46	6.72	6.59	3.80
Tyr158	16.85	16.22	14.42	15.86	15.70	15.76	17.09	16.61	10.00
Lys166	10.16	9.84	10.00	10.32	9.99	10.03	10.05		
Lys180	8.71	8.48	8.88	8.92	8.60	8.42	8.49	8.75	10.50
Asp218	3.00	3.30	2.98	3.26	2.71	2.93	3.16	3.18	3.80
Glu255	7.34	4.01	6.09	3.87	7.69	8.48	8.03	8.95	4.50
Cys289	N/A	N/A	13.43	14.70	N/A	N/A	N/A	14.14	9.00
Lys339	10.45	8.73	10.91	10.84	10.03	10.19	9.92	10.44	10.50
Glu390	7.01	6.77	4.81	7.92	7.77	7.26	5.58	4.97	4.50
His448	4.37	4.43	4.44	3.66	4.14	4.50	4.23	4.38	6.50
Tyr450	15.17	14.74	15.90	17.34	10.09	15.16	14.94	15.41	10.00
Glu467	4.77	4.97	4.72	4.86	4.82	4.82	4.41	5.26	4.50
NAD									
N7A	N/A	3.73	N/A	3.87	N/A	N/A	N/A	3.72	5.00
O1A	N/A	6.56	N/A	7.57	N/A	N/A	N/A	7.25	6.00
O2N	N/A	7.90	N/A	7.69	N/A	N/A	N/A	8.61	6.00
pH of a protein sample	8.3								
pH of crystallization	8.5	8.5	8.5	N/A	3.5	8.0	8.0	8.5	

[a]Chain *A* of each PDB entry was used only.

Supporting Information

Structural and functional analysis of betaine aldehyde dehydrogenase from Staphylococcus aureus

Halavaty et al.

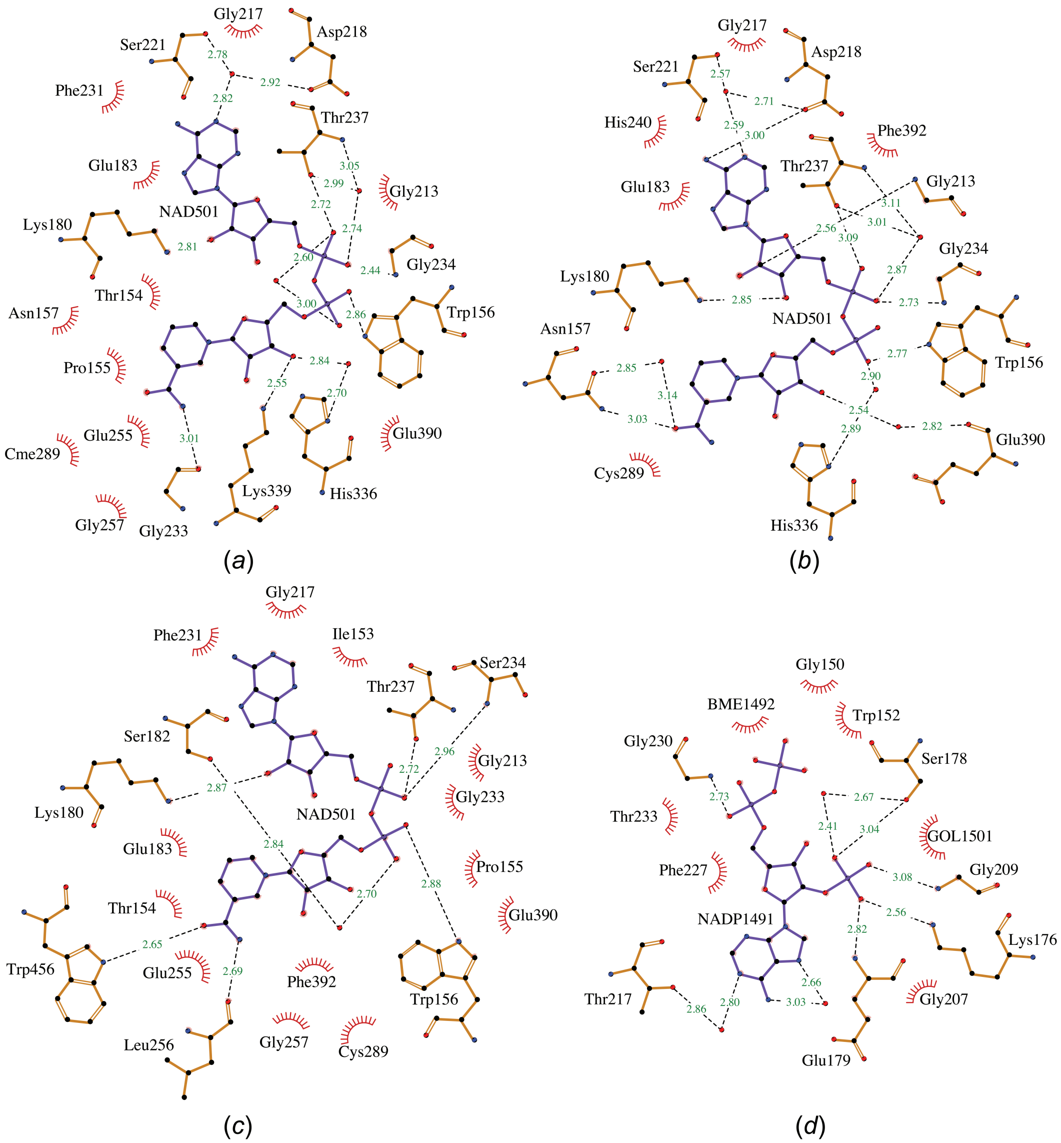

Figure S1. Details of the NAD–*Sa*BADH interactions in NAD–*Sa*BADH$^{BME(-)}$ (*a*), NAD–*Sa*BADH$^{BME(+)}$ (*b*) and NAD–G234S-*Sa*BADH$^{BME(+)}$ (*c*). (*d*) Coordination of NADP^{+} in the *Pa*BADH structure (PDB entry 2wme). The NMN moiety was not modelled

in the 2wme structure and, thus, is not displayed. BME – β-mercaptoethanol; GOL – glycerol. Cme289 is BME-modified Cys289. Water molecules are red spheres.
